# ACOD1 deficiency offers protection in a mouse model of diet-induced obesity by maintaining a healthy gut microbiota

**DOI:** 10.1038/s41419-024-06483-2

**Published:** 2024-02-01

**Authors:** Tanja Eberhart, Federico Uchenna Stanley, Luisa Ricci, Tiziana Chirico, Roberto Ferrarese, Sofia Sisti, Alessandra Scagliola, Andreina Baj, Sylvia Badurek, Andreas Sommer, Rachel Culp-Hill, Monika Dzieciatkowska, Engy Shokry, David Sumpton, Angelo D’Alessandro, Nicola Clementi, Nicasio Mancini, Simone Cardaci

**Affiliations:** 1 Cancer Metabolism Unit, Division of Genetics and Cell Biology, IRCCS San Raffaele Scientific Institute, 20132 Milan, Italy; 2 Laboratory of Medical Microbiology and Virology, Vita-Salute San Raffaele University, Milan, 20100 Italy; 3 IRCCS San Raffaele Hospital, Milan, 20100 Italy; 4 Department of Medicine and Technological Innovation, University of Insubria, Varese, Italy; 5 Preclinical Phenotyping Facility, Vienna BioCenter Core Facilities (VBCF), member of the Vienna BioCenter (VBC), Vienna, Austria; 6 Next Generation Sequencing Facility, Vienna BioCenter Core Facilities (VBCF), member of the Vienna BioCenter (VBC), Vienna, Austria; 7 Department of Biochemistry and Molecular Genetics, Anschutz Medical Campus, University of Colorado School of Medicine, Aurora, CO 80045 USA; 8 CRUK Beatson Institute, Glasgow, UK; 9 Present Address: Synlab Italia, Castenedolo, BS Italy; 10 Present Address: Istituto Nazionale di Genetica Molecolare, INGM, “Romeo ed Enrica Invernizzi”, Milan, Italy; 11 Present Address: Laboratory of Medical Microbiology and Virology, Department of Medicine and Technological Innovation, University of Insubria, Varese, Italy; 12 Present Address: Laboratory of Medical Microbiology and Virology, Fondazione Macchi University Hospital, Varese, Italy

**Keywords:** Obesity, Dysbiosis

## Abstract

Aconitate decarboxylase 1 (ACOD1) is the enzyme synthesizing itaconate, an immuno-regulatory metabolite tuning host-pathogen interactions. Such functions are achieved by affecting metabolic pathways regulating inflammation and microbe survival. However, at the whole-body level, metabolic roles of itaconate remain largely unresolved. By using multiomics-integrated approaches, here we show that ACOD1 responds to high-fat diet consumption in mice by promoting gut microbiota alterations supporting metabolic disease. Genetic disruption of itaconate biosynthesis protects mice against obesity, alterations in glucose homeostasis and liver metabolic dysfunctions by decreasing meta-inflammatory responses to dietary lipid overload. Mechanistically, fecal metagenomics and microbiota transplantation experiments demonstrate such effects are dependent on an amelioration of the intestinal ecosystem composition, skewed by high-fat diet feeding towards obesogenic phenotype. In particular, unbiased fecal microbiota profiling and axenic culture experiments point towards a primary role for itaconate in inhibiting growth of *Bacteroidaceae* and *Bacteroides*, family and genus of *Bacteroidetes* phylum, the major gut microbial taxon associated with metabolic health. Specularly to the effects imposed by *Acod1* deficiency on fecal microbiota, oral itaconate consumption enhances diet-induced gut dysbiosis and associated obesogenic responses in mice. Unveiling an unrecognized role of itaconate, either endogenously produced or exogenously administered, in supporting microbiota alterations underlying diet-induced obesity in mice, our study points ACOD1 as a target against inflammatory consequences of overnutrition.

## Introduction

Obesity is a chronic condition of excessive fat accumulation associated with a body mass index (BMI) ≥ 30 kg/m^2^. With over 13% of the world’s population affected, obesity represents a global health concern, as it predisposes individuals to the development of chronic diseases including type 2 diabetes, non-alcoholic fatty liver disease (NAFLD) and several cancer types [1, 2].

Obesity develops when chronic energy intake exceeds energy expenditure, causing the accumulation of unused nutrients as fat in adipose tissue depots. The resulting adipocyte hypertrophy initiates a chronic low-grade inflammation (also known as meta-inflammation), characterized by production of pro-inflammatory mediators by adipocytes as well as adipose tissue-resident and recruited innate and adaptive immune cells [3, 4]. In turn, the engagement of meta-inflammatory networks within fat depots impairs adipocyte liporegulatory capacities, inhibits energy-expenditure, including adaptive thermogenesis in brown adipose tissue (BAT) and white adipose tissue (WAT) browning, as well as propagates major obesity-associated sequelae, such as decreased sensitivity to insulin and ectopic lipid deposition [3, 4]. In both rodents and humans, obesity is associated with gut microbiota imbalance (dysbiosis), mainly due to a decreased proportion between *Bacteroidetes* and *Firmicutes*, the two largest bacterial phyla of the intestinal ecosystem [5–7]. Intestinal dysbiosis participates in metabolic disease by stimulating food intake and energy extraction from the consumed diet as well as by promoting meta-inflammation in the host through decreased gut barrier function and altered microbial synthesis of signaling metabolites influencing host inflammatory responses, such as short-chain fatty acids (SCFA) [8, 9].

Metabolic changes in cells shape intestinal microbiota composition and regulate meta-inflammatory responses in obesity [10, 11]. Itaconate is a metabolite synthesized from the tricarboxylic acid (TCA) cycle intermediate *cis*-aconitate, catalyzed by the enzyme aconitate decarboxylase 1 (ACOD1) in animals [12]. Although poorly detectable in resting conditions, ACOD1 and itaconate levels are upregulated in immune (mainly macrophages) and non-immune cells in response to pro-inflammatory and oxidative conditions to sustain immunoregulatory and antioxidant programs [13–15]. Furthermore, itaconate biosynthesis is induced in cells following infections to regulate host-pathogen interactions [16]. The generation of an antiviral metabolic state in virus-infected cells and the inhibition of pro-survival bacterial metabolic pathways, such as the glyoxylate shunt and methylcitrate cycle, mainly account for antimicrobial roles of itaconate and its natural derivatives [12, 17, 18]. However, several bacteria can benefit from host-produced itaconate either by using it as a carbon source or as a signal to rewire their metabolism to promote biofilm formation [19, 20]. Aligned with this, mounting evidence points toward an instrumental role for the itaconate biosynthesis machinery in driving bacterial replication and pro-inflammatory lethal immune responses to microbial sepsis [16, 21].

Although its impact on cell metabolism has been widely investigated, understanding the role of itaconate biosynthesis in whole-body metabolic responses to nutritional perturbations remains largely unresolved. Here, we demonstrate that ACOD1 responds to dietary lipid overload by promoting gut microbiota alterations supporting obesity and its major inflammatory outcomes. *Acod1* loss opposes glycemic homeostasis alterations, liver steatosis, and associated urea cycle dysfunction as well as dampens meta-inflammation signatures induced by overnutrition in mice. Fecal metagenomics profiling and microbiota transplantation experiments demonstrate such protection is dominant, transferable and results from an amelioration of the intestinal ecosystem, altered by high-fat diet consumption, pointing towards itaconate biosynthesis as a novel target for intervention against metabolic disease.

## Results

### Genetic *Acod1* deficiency protects mice from diet-induced obesity

**Fig. 1:**
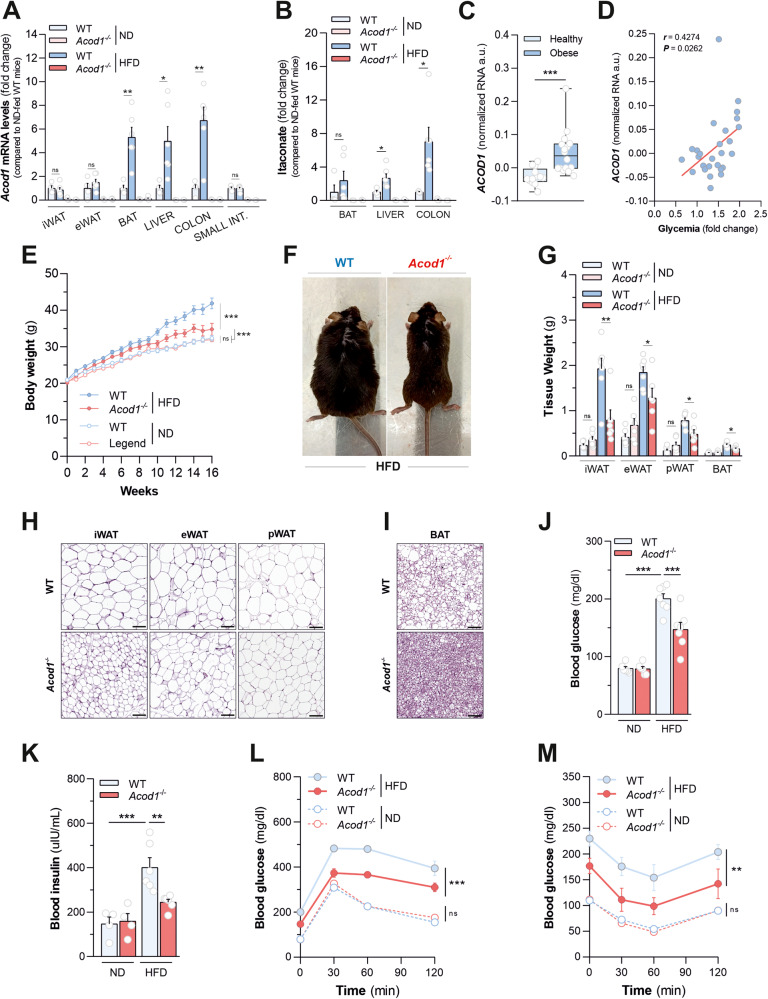
Acod1 deficiency protects mice from diet-induced obesity. **A** qPCR analysis of *Acod1* mRNA levels in the indicated tissues from WT and *Acod1*^*-/-*^ mice fed with ND (*n* = 4) or HFD (*n* = 6) for 16 weeks. Data as mean ± sem from one experiment out of two independently executed. **B** Itaconate levels in indicated tissues from mice as described in **A**; ND (*n* = 4), HFD-WT (*n* = 6), HFD-*Acod1*^*-/-*^ (*n* = 4). Data as mean ± sem from one experiment out of two independently executed. **C** *ACOD1* mRNA levels (GSE158237) in colon of healthy (BMI < 25 kg/m^2^, *n* = 13) and obese subjects (BMI > 30 kg/m^2^, *n* = 16). Boxes extend from 25th to 75th percentiles, black lines = median, whiskers=minimum to maximum. **D** Correlation analysis between colonic *ACOD1* mRNA (GSE158237) and relative changes in plasma glucose levels measured 2 h after oral glucose overload (OGTT) compared with baseline, in human subjects described in Fig. 1C. *r* = Pearson’s correlation coefficient. **E** Body weights of mice as described in **A**. Data as mean ± sem of *n* = 17 (ND-WT), *n* = 18 (ND- *Acod1*^*-/-*^), *n* = 26 (HFD-WT) and *n* = 22 (HFD-*Acod1*^*-/-*^) from three experiments independently executed. **F** Representative images of WT and *Acod1*^*-/-*^ mice after HFD. **G** Weights (g) of the indicated tissues from mice treated as in **A** at the end of the experiment; ND (*n* = 6), HFD-WT (*n* = 7), HFD-*Acod1*^*-/-*^ (*n* = 6). Data as mean ± sem from one representative experiment out of three independently executed. Representative images of H&E staining of iWAT, eWAT, pWAT (**H**) and BAT (**I**) from HFD-fed WT and *Acod1*^*-/-*^ mice. **J** Fasting glycemia of mice treated as in **A**; ND (*n* = 6), HFD (*n* = 7). Data as mean ± sem from one representative experiment out of two independently executed. **K** Fasting insulinemia of in mice treated as in **A**; ND (*n* = 4), HFD-WT (*n* = 7), HFD-*Acod1*^*-/-*^ (*n* = 6). Data as mean ± sem from one representative experiment out of two independently executed. **L** Glucose tolerance test (GTT) in mice treated as in **A**; ND (*n* = 6), HFD (*n* = 7). Data as mean ± sem from one representative experiment out of two independently executed. **M** Insulin tolerance test (ITT) in mice described in **A**; ND (*n* = 5), HFD (*n* = 7). Data as mean ± sem from one representative experiment out of two independently executed. In: (**A**–**C**, **G**): t-test; (**D**) Pearson correlation; (**E**), (**L**), and (**M**) two-way ANOVA; (J-K), one-way ANOVA with Dunnett’s multiple comparisons test compared to HFD-fed WT mice. *P < 0.05; **P < 0.01; ***P < 0.001; ns not significant.

*Acod1* is a gene with barely detectable baseline expression but inducible on stress. To determine whether *Acod1* is responsive to fat overnutrition, we challenged five-week old wild type mice with standardized high-fat diet (HFD) containing 60% of calories from fat for 16 weeks to promote metabolic disease. Time-resolved profiling of *Acod1* mRNA levels in several tissues involved in responses to dietary lipid overload revealed increased gene expression in colon starting from 12 weeks of HFD administration, as well as brown adipose tissue (BAT) and liver after additional 4 weeks, with respect to age-matched littermates fed with normal diet (ND) (Fig. 1A and Supplementary Fig. 1A). Furthermore, a statistical significant increase of itaconate levels was detected in liver and colon of mice following HFD consumption, matching the corresponding timing of *Acod1* gene expression induction in both tissues (Fig. 1B and Supplementary Fig. 1B).

*Acod1* is known to be prevalently expressed in macrophages. Results shown in Supplementary Fig. 1C indicate that HFD consumption does not result in a significant increase of the major macrophage marker F4/80 (*Adgre1*) mRNA levels (used as a proxy for macrophage accumulation) in BAT of wild type mice throughout the experiment. On the contrary, a significant accumulation of F4/80 mRNA was observed in liver and colon of HFD-fed mice only at 16 weeks of HFD feeding. These data, combined with the time-resolved profiling of *Acod1* mRNA levels in the same tissues (Fig. 1A and Supplementary Fig. 1A), suggest that the increase of *Acod1* mRNA in colon and BAT might result from a true up-regulation of its gene expression, whereas it might be considered just a proxy for increased macrophage accumulation in the liver. Querying for translational extension of data generated in mice, we retrieved *ACOD1* expression in publicly available transcriptomic datasets of human subjects with obesity and the associated NAFLD (Supplementary Table 1)*. ACOD1* mRNA levels were found higher in colon biopsies of obese subjects with respect to healthy counterparts (Fig. 1C) and correlated with lower tolerance to oral glucose overload (Fig. 1D). Also, increased *ACOD1* expression was found in livers of NAFLD patients in dependency of disease severity (Supplementary Fig. 1D) and hepatic steatosis grades (Supplementary Fig. 1E).

Then, to understand the role of ACOD1 in the whole-body responses to fat overnutrition we challenged *Acod1* knockout (*Acod1*^-/-^) mice with either standard chow or HFD and compared their responses to wild-type counterparts on the same dietary regimen. Genetic ablation of *Acod1* abrogated detection of its mRNA levels (Fig. 1A) and HFD-induced itaconate accumulation in tissues (Fig. 1B). Unexpectedly, we found *Acod1*^-/-^ mice gained less weight than their wild-type littermates, on HFD (Fig. 1E, F). Consistent with this, *Acod1* loss resulted in lower accumulation of adiposity in response to dietary lipid overload, as demonstrated by a considerable decrease in weight of inguinal (iWAT), epididymal (eWAT), and perirenal (pWAT) white fat depots as well as BAT measured in HFD-fed *Acod1*^*-/-*^ mice, compared with wild type counterparts (Fig. 1G), without any change in the mass of several lean soft tissues (Supplementary Fig. 1F). In support of such evidence, significant decreases in white adipocyte size (Fig. 1H and Supplementary Fig. 1G) and BAT whitening (Fig. 1I and Supplementary Fig. 1H) were detected by histomorphometry of fat depots isolated from *Acod1*^*-/-*^ mice challenged with HFD, with respect to wild type controls. Consistent with the lower fat mass, HFD-fed *Acod1*^*-/-*^ mice displayed lower fasting glycemia (Fig. 1J) and insulinemia than wild type mice (Fig. 1K). In line, improved responses to intraperitoneal glucose overload (Fig. 1L and Supplementary Fig. 1I) and insulin administration (Fig. 1M and Supplementary Fig. 1J), were imposed by *Acod1* ablation in HFD-fed mice, without changes in GLP-1 and resistin, hormones regulating sensitivity to insulin (Supplementary Fig. 1K). On the contrary, on a regular chow, *Acod1*^-/-^ mice did not show any overt phenotype maintaining overall body weight (Fig. 1E), adiposity (Fig. 1G), fasting blood glucose (Fig. 1J) and insulin levels (Fig. 1K), glucose tolerance (Fig. 1L and Supplementary Fig. 1I**)** and insulin sensitivity (Fig. 1M and Supplementary Fig. 1J) comparable to those of age-matched wild type counterparts. Importantly, oral administration of itaconate (1 mM) to HFD-fed *Acod1*^*-/-*^ mice was sufficient to rescue the levels of itaconate accumulation in the colon (Supplementary Fig. 2A) and to restore the effects of gene loss on body weight gain (Supplementary Fig. 2B), adiposity (Supplementary Fig. 2C) and insulin resistance (Supplementary Fig. 2D). Collectively, these results demonstrate that deficiency of ACOD1 activity in mice offers an obvious amelioration - although not a complete abrogation - of diet-induced obesity and the associated alterations of glucose homeostasis.

### *Acod1* ablation opposes liver steatosis and associated urea cycle dysfunction induced by dietary lipid overload

**Fig. 2:**
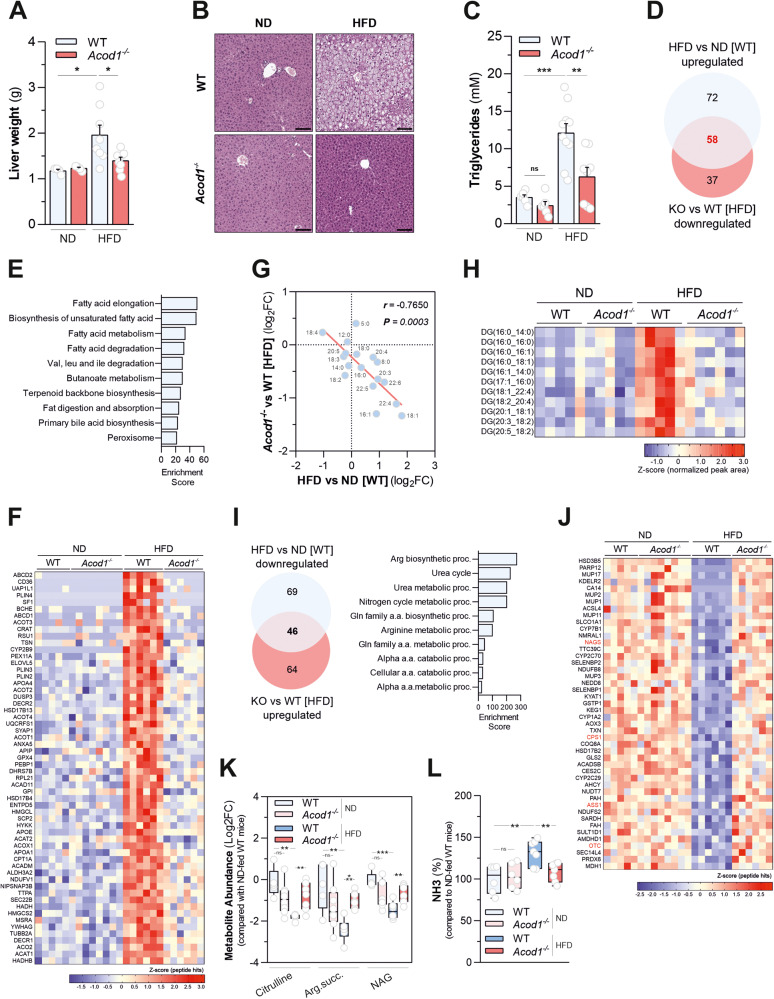
*Acod1* ablation opposes liver steatosis and associated urea cycle dysfunction induced by dietary lipid overload. **A** Weights of livers from WT and *Acod1*^*-/-*^ mice fed with ND (*n* = 4) or HFD (*n* = 8) for 16 weeks. Data as mean±s.e.m. from one experiment out of three independently executed. **B** Representative H&E staining of livers from mice described in **A**, Bar = 100 μm. **C** Liver triglycerides in mice described in **A**; ND (*n* = 6), HFD-WT (*n* = 10), HFD-*Acod1*^*-/-*^ (*n* = 8). Data as mean±s.e.m. from two independent experiments. Venn diagram (**D**) and KEGG-based functional enrichment analysis (**E**) of upregulated proteins in livers of WT mice fed with HFD compared with ND-fed mice (HFD vs ND [WT]) and rescued by *Acod1* loss (*Acod1*^*-/-*^ vs WT [HFD]). Data derive from *n* = 5 ND-fed (WT), *n* = 8 (ND-fed *Acod1*^*-/-*^) and *n* = 6 (HFD-fed) mice from two independent experiments. (**F**) Heatmap of protein levels described in **D**. **G** Pearson’s correlation between changes in liver free fatty acid (FFA) levels in HFD-fed WT mice (HFD vs ND [WT]) and by *Acod1* loss (*Acod1*^*-/-*^ vs WT [HFD]); *n* = 6 pre group. Data from two independent experiments. **H** Heatmap depicting diacylglycerol levels in livers of mice treated as in **A**. Data derive from *n* = 5 (ND-fed), *n* = 5 (HFD-fed WT), *n* = 6 (HFD-fed *Acod1*^*-/-*^) mice from one experiment. **I** Venn diagram (left) and gene ontology (biological processes)-based functional enrichment analysis (right) of downregulated proteins in livers of WT mice fed with HFD compared with ND-fed mice (HFD vs ND [WT]) and rescued by *Acod1* loss (*Acod1*^*-/-*^ vs WT [HFD]). **J** Heatmap of protein levels described in **I**. **K** Levels of the indicated urea cycle metabolites in liver of mice fed with either ND or HFD for 16 weeks. Data derive from *n* = 5 (ND-fed wild type), *n* = 8 (ND-fed *Acod1*^*-/-*^), *n* = 6 (HFD-fed wild type), and *n* = 7 (HFD-fed *Acod1*^*-/-*^) mice from two independent experiments; Arg.succ argininosuccinate, NAG *N*-Acetylglutamate. **L** Levels of NH_3_ in liver of mice fed with either ND or HFD for 16 weeks. Data derive from *n* = 6 (ND), *n* = 7 (HFD)-fed mice from two independent experiments. In **K** and **L**, boxes extend from 25th to 75th percentiles, black lines=median, whiskers=minimum to maximum. In (**A**) one-way ANOVA with Dunnett’s multiple comparisons test compared to HFD-fed WT mice; In **C**, (**K**, **L**): *t*-test. *P < 0.05; **P < 0.01; ***P < 0.001; ns not significant.

Diet-induced obesity might result in lipid deposition in the liver, setting the stage for NAFLD development. Necroscopic analyses indicated that livers isolated from HFD-fed wild type mice were bigger (Fig. 2A) with respect to *Acod1*^-/-^ counterparts. Histological examination of liver sections (Fig. 2B) and quantitative determination of hepatic triglyceride content (Fig. 2C) clearly showed that *Acod1* loss attenuated HFD-induced liver steatosis. On the contrary, livers isolated from *Acod1*^-/-^ mice displayed similar weight and fat content with respect to their controls, on a regular chow (Fig. 2A–C). Next, to gain deeper insights into the impact of *Acod1* deficiency on hepatic responses to HFD, we compared livers from wild type and *Acod1*^-/-^ mice fed with standard chow or HFD by untargeted label-free quantitative proteomics. Principal component analysis showed an obvious separation between the hepatic proteome of mice challenged with HFD compared with their controls (Supplementary Fig. 3A). Moreover, the occurrence of a sub-clustering between HFD-challenged wild type and *Acod1*^-/-^ mice, but not in their corresponding normal diet-fed controls, indicated that *Acod1* loss generates a molecular signature to fat overnutrition. Specifically, *Acod1* deficiency opposed the impact of dietary lipid overload on the hepatic proteome, as demonstrated by the significant negative correlation computed between changes in protein abundances occurring in liver of wild-type mice upon HFD and those determined by *Acod1* loss on the same dietary regimen (Supplementary Fig. 3B). In particular, the levels of 130 proteins were significantly increased in liver of HFD-challenged wild type mice compared with normal diet-fed controls and the content of almost half of them (58) were significantly down-regulated by *Acod1* loss (Fig. 2D). Functional annotation analysis revealed that the restored proteome was mainly enriched with metabolic categories related to fatty acid biosynthesis and processing pathways (Fig. 2E and Supplementary Table 2). Consistent with the amelioration of the steatotic phenotype, *Acod1* loss counteracted the HFD-induced hepatic accumulation of perilipins (PLIN2, PLIN3, PLIN4), which sequester lipids by protecting lipid droplets from lipase action, enzymes responsible for peroxisomal fatty acid β-oxidation (ACOX1, HSD17B4, SCP2, CRAT, ACOT2, ACOT4, DECR2), known to promote HFD-induced liver steatosis by inhibiting hepatic lipophagy [22], as well as CD36 which promotes fatty liver development by facilitating free fatty acid (FFAs) uptake in hepatocytes [23, 24] (Fig. 2F). In particular, the attenuation of CD36 content was associated with decreased abundance of several FFAs (Fig. 2G) and their corresponding diacylglycerol species (Fig. 2H), accumulating in liver of mice following HFD consumption. Comparative proteomics also retrieved 46 entities whose levels were significantly decreased in liver of wild-type mice following HFD challenge and restored by *Acod1* gene ablation (Fig. 2I and Supplementary Table 2). Strikingly, four of them, ornithine transcarbamylase (OTC), carbamoyl phosphate synthetase 1 (CPS1), argininosuccinate synthetase 1 (ASS1), and *N*-acetylglutamate synthase (NAGS) (Fig. 2J) are enzymes involved in the urea cycle, a metabolic route responsible for ammonia detoxification impaired in NAFLD experimental models and patients, as a result of hepatic fatty acid accumulation [25], contributing to development of hepatic fibrosis [26]. In line with protection from diet-induced liver steatosis, functional enrichment analysis ranked urea cycle as the most significantly altered pathway by HFD regimen and rescued in *Acod1*^*-/-*^ mice (Fig. 2I). The changes in the levels of such proteins imposed by genotype occurred mainly following HFD administration and not upon normal chow (Fig. 2J) and were associated with alterations in the expression of their encoding genes (Supplementary Fig. 3C). Moreover, the emergence of such proteomic signature induced by HFD consumption was linked to decreased levels of associated urea cycle metabolites and accumulation of ammonia in the liver, both restored by *Acod1* loss (Fig. 2K, L). Overall, these data indicate that *Acod1* deficiency protects mice from hepatic steatosis and the NAFLD-associated urea cycle dysfunction induced by fat overfeeding.

### Acod1 loss improves metabolic activity of HFD-fed mice

**Fig. 3:**
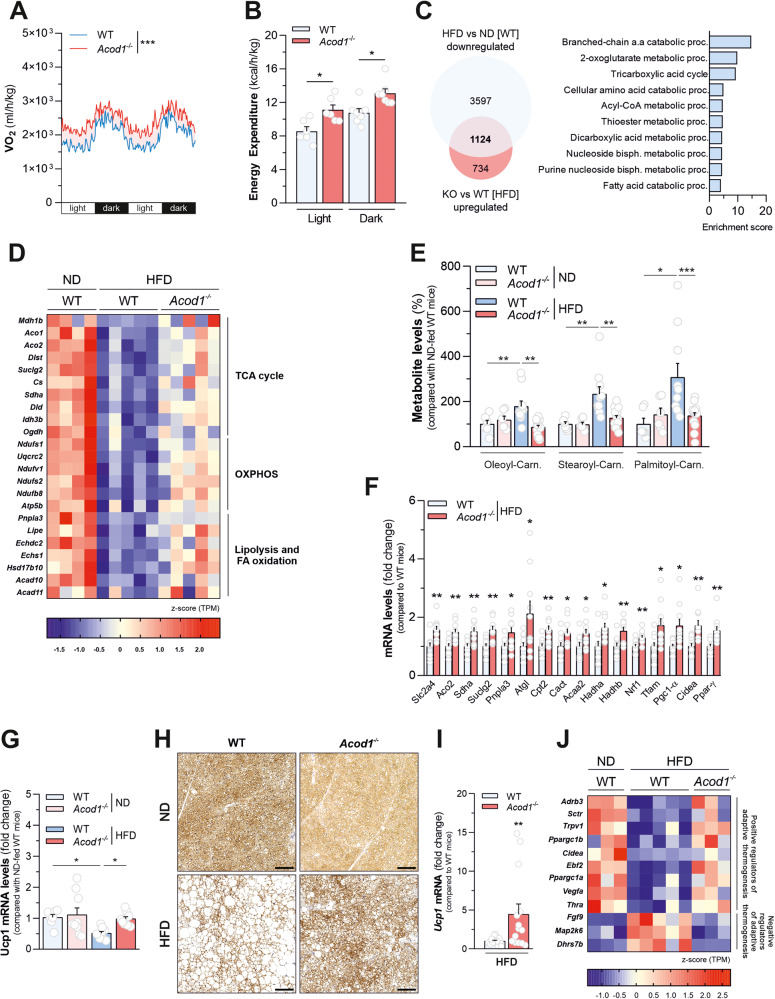
*Acod1* loss improves metabolic activity of HFD-fed mice. Whole-body O_2_ consumption rate (VO_2_) (**A**) and energy expenditure rate (**B**) normalized on body weight of HFD-fed WT (*n* =*n* = 6) and *Acod1*^*-/-*^ (*n* = 8) mice during light and dark hours (one representative experiment out of two independently executed). **C** Venn diagram (left) and gene ontology (biological processes)-based functional enrichment analysis (right) of downregulated proteins in eWAT of WT mice fed with HFD for 16 weeks compared with ND-fed mice (HFD vs ND [WT]) and rescued by *Acod1* loss (*Acod1*^*-/-*^ vs WT [HFD]). Data derive from *n* = 4 (ND-fed WT) and *n* = 5 (HFD-fed) mice from one experiment. **D** Heatmap depicting mRNA levels of selected genes related to tricarboxylic acid (TCA) cycle, oxidative phosphorylation (OXPHOS), lipolysis and fatty acid (FA) oxidation downregulated in eWAT of HFD-fed WT mice (HFD vs ND [WT]) and rescued by *Acod1* loss (*Acod1*^*-/-*^ vs WT [HFD]) Data derive from mice described in **C**: *n* = 4 (ND-fed WT) and *n* = 5 (HFD-fed) mice from one experiment. **E** Acyl-carnitine levels in iWAT of mice fed with either ND (*n* = 6) or HFD (*n* = 10) from two independent experiments. Carn.: carnitine (**F**) qPCR analysis of mRNA levels of the indicated genes in BAT of mice described in **A**; *n* = 10 per group from two independent experiments. **G** qPCR analysis of *Ucp1* mRNA levels in BAT of WT and *Acod1*^*-/-*^ mice fed with either ND or HFD for 16 weeks. *n* = 7–9 per group from two independent experiments. **H** Representative images of UCP1 immunohistochemical staining normalized to cell number in BAT of mice described in **G**. Bar = 100 μm. **I** qPCR analysis of *Ucp1* mRNA levels in iWAT of mice described in **A**; WT (*n* = 15), *Acod1*^*-/-*^ (*n* = 14) mice from three independent experiments. **J** Heatmap depicting mRNA levels of selected genes known as inducers (positive regulators) or inhibitors (negative regulators) of adaptive thermogenesis changed in iWAT of HFD-fed WT mice and rescued by *Acod1* loss. Data derive from *n* = 3 (ND-fed WT), *n* = 5 (HFD-fed WT), and *n* = 3 (HFD-fed *Acod1*^*-/-*^) mice from one experiment. In: (**B**), (**E**, **F**), (**G**, **I**) data as mean±s.e.m; (**A**) two-way ANOVA; (**E**, **G**) one-way ANOVA followed by Dunnett’s multiple comparisons test compared to HFD-fed WT mice; (**B**, **F**, **I**), *t* test; *P < 0.05; **P < 0.01; ***P < 0.001.

The leaner phenotype observed in HFD-challenged *Acod1*^-/-^ mice did not result from reduction in food consumption (Supplementary Fig. 4A) or impairment of intestinal nutrient absorption, evaluated by bomb calorimetry-mediated measurement of fecal energy content (Supplementary Fig. 4B). Furthermore, it was not associated with genotype-dependent perturbations of the basal adipogenic potential, assessed by measuring neutral lipid accumulation and mRNA levels of key adipogenic regulators in cultured primary iWAT pre-adipocytes, isolated from wild type and *Acod1*^-/-^ mice, upon differentiation stimulation (Supplementary Fig. 4C, D). Therefore, to elaborate on the mechanisms responsible for resistance to diet-induced obesity and the associated metabolic dysfunctions promoted by *Acod1* deficiency, we performed whole-body metabolic analyses of HFD-fed mice by indirect calorimetry. A significant increase in oxygen consumption (Fig. 3A) and energy expenditure (Fig. 3B), during both light and dark phases, were observed in *Acod1*^-/-^ mice compared with wild-type littermates. Such results demonstrate that *Acod1* loss imposes a higher metabolic rate upon HFD challenge, not dependent on changes in preferential substrate use, as indicated by the unaffected respiratory exchange ratio (Supplementary Fig. 4E). Additionally, such changes were neither associated with genotype-dependent alterations of circulating leptin levels (Supplementary Fig. 4F) nor attributable to changes in spontaneous locomotor activity (Supplementary Fig. 4G). Next, to determine whether fat tissues contributed to the higher energy expenditure in overfeeding conditions, we profiled gene expression changes determined by *Acod1* loss in eWAT isolated from HFD-fed mice by RNAseq. Mitochondrial dysfunction and compromised oxidative metabolism are considered hallmarks of obese adipose tissue [27]. In line with this and the enhanced metabolic activity detected in HFD-fed *Acod1*^-/-^ mice, the transcriptional profile of genes significantly down-regulated in eWAT of wild-type mice in response to HFD and rescued by *Acod1* loss was mainly enriched with functional categories defining oxidative metabolic processes and mitochondrial energy-producing pathways (Fig. 3C and Supplementary Table 3). In particular, *Acod1* loss opposed the HFD-induced transcriptional suppression of genes encoding TCA cycle enzymes (*Mdh1b*, *Aco1*, *Dlst*, *Suclg2*, *Cs*, *Sdha*, *Dld*, *Idh3b*, *Ogdh*) as well as components of mitochondrial electron transport chain (*Ndufs1, Uqcrc2, Ndufv1, Ndufb8, Atp5b*) (Fig. 3D). Consistent with this, increased mRNA levels of the transcriptional coactivator *Pgc-1α* (*Ppargc1a*), a master regulator of mitochondrial oxidative metabolism, were observed in HFD-fed *Acod1*^-/-^ mice, compared with wild type counterparts (Supplementary Fig. 4H). Furthermore, *Acod1* ablation rescued the mRNA levels of genes involved in neutral lipolysis (*Pnpla3* and *Lipe*) and mitochondrial fatty acid oxidation (*Echdc2*, *Echs1*, *Hsd17b10*, *Acad10*, *Acad11*) (Fig. 3D), downregulated in adipose tissue of wild type mice in response to fat overfeeding. It is worth noting that acyl-carnitine accumulation occurs when fatty acid supply exceeds mitochondrial β-oxidation and TCA cycle capacity [28]. In line with this and the rescue of lipid catabolic genes, *Acod1* loss normalized the levels of major long-chain acyl-carnitines (palmitoyl-, oleoyl-, stearoyl-carnitine), increased in eWAT of wild type mice following dietary lipid overload (Fig. 3E). BAT contributes to energy expenditure by engaging catabolic processes sustaining non-shivering (adaptive) thermogenesis, mainly mediated by uncoupling protein 1 (UCP1) activity [29]. Furthermore, WAT browning might participate in energy dissipation, thereby counteracting obesity manifestation [30, 31]. Consistent with this, increased mRNA levels of representative genes promoting triglyceride and fatty acid catabolism (*Pnpla3*, *Atgl*, *Cpt2*, *Cact*, *Acaa2*, *Hadha*, *Hadhb*) mitochondrial biogenesis (*Nrf1*, *Tfam*, *Pgc1-α*) and involved in central carbon metabolism (*Slc2a4*, *Aco2*, *Sdha*, *Suclg2*) were detected in BAT collected from HFD-fed *Acod1*^-/-^ mice, compared with wild type controls (Fig. 3F). Furthermore, *Acod1* loss resulted in increased expression of genes for non-shivering thermogenesis, such as *Ucp1* (Fig. 3G), *Ppar-γ* and *Cidea* (Fig. 3F) as well as stronger UCP1 immunostaining (Fig. 3H and Supplementary Fig. 4I) in BAT isolated from HFD-fed mice. Similarly, *Acod1* ablation resulted in up-regulation of *Ucp1* mRNA levels in iWAT isolated from HFD-fed mice (Fig. 3I) and opposed the changes in the expression of several genes regulating the adaptive thermogenic response, elicited by fat overfeeding in wild type mice, retrieved by RNAseq (Fig. 3J and Supplementary Table 4). On the contrary, no alterations in the mRNA levels of representative genes involved in fatty acid catabolism and oxidative mitochondrial metabolism were imposed by genotype in quadriceps (Supplementary Fig. 4J) and tibialis anterior (Supplementary Fig. 4K) - chosen as representative lean mass tissues - isolated from HFD-fed mice. Collectively, these results indicate *Acod1* ablation improves oxidative efficiency in conditions of fat overnutrition, mainly associated with increased catabolic signatures in adipose tissues.

### Acod1 loss decreases diet-induced obesity-associated meta-inflammation

**Fig. 4:**
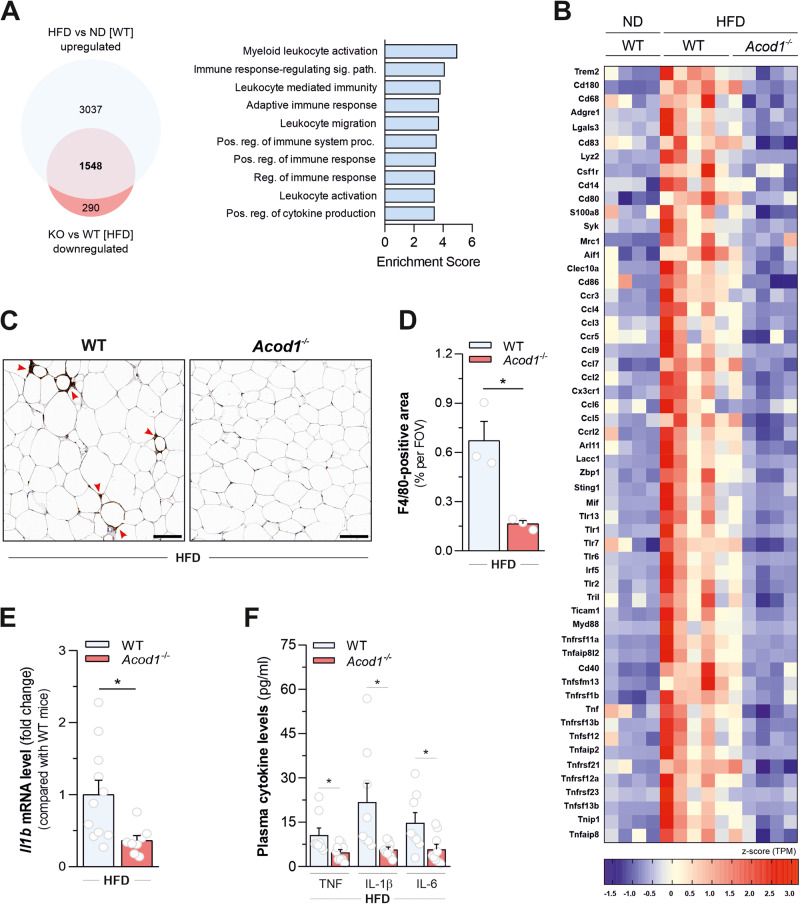
*Acod1* loss decreases diet-induced obesity-associated meta-inflammation. **A** Venn diagram (left) and gene ontology (biological processes)-based functional enrichment analysis (right) of upregulated genes in eWAT of WT mice fed with HFD for 16 weeks compared with ND-fed mice (HFD vs ND [WT]) and rescued by *Acod1* loss (*Acod1*^*-/-*^ vs WT [HFD]). Data derive from *n* = 4 (ND-fed WT) and *n* = 5 (HFD-fed) mice from one experiment. **B** Heatmap depicting the mRNA levels of selected genes defining macrophage markers, inflammatory cytokines and receptors, regulators of innate immunity, TLR signaling and TNF signaling upregulated in response to HFD (HFD vs ND [WT], FDR < 0.1) and rescued by *Acod1* loss (*Acod1*^*-/-*^ vs WT [HFD], FDR < 0.1) in eWAT. Data derive from mice described in **A**: *n* = 4 (ND-fed WT) and *n* = 5 (HFD-fed) mice from one experiment. **C** Representative images (left) of immunohistochemistry staining for the macrophage marker F4/80 (*Adgre1*) in eWAT of WT and *Acod1*^-/-^ mice after 16 weeks on HFD diet. Red arrows indicate crown-like structures. **D** Quantification of the F4/80-positive area per field of view (FOV) in images described in **C**. (*n* = 5 mice per group, average of 5 images each). Red arrows indicate crown-like structures. Bars, 100 μm. Data are represented as mean ± s.e.m. *P < 0.05 (*t*-test). **E** q*P*CR analysis of *Il1b* mRNA levels in BAT of WT and *Acod1*^*-/-*^ mice after 16 weeks on HFD diet. Data as as mean±s.e.m of *n* = 11 (WT) and *n* =*n* = 8 (*Acod1*^*-/-*^) mice from two independent experiments. *P < 0.05 (*t*-test). **F** Levels of the indicated pro-inflammatory cytokines in plasma of WT and *Acod1*^*-/-*^ mice after 16 weeks on HFD diet. Data as as mean±s.e.m of *n* = 8 (WT) and *n* = 9 (*Acod1*^*-/-*^) mice from two independent experiments. *P < 0.05 (*t*-test).

Obesity is associated with adipose tissue macrophage infiltration [32] promoting chronic low-grade inflammation [33], contributing to impaired insulin sensitivity and energy expenditure processes [4]. We confirmed that ACOD1 loss enhances pro-inflammatory cytokine production in bone marrow-derived macrophages (BMDMs) challenged in vitro with LPS (Supplementary Fig. 5A–C). However, decreased leukocyte activation and inflammatory responses were revealed by functional analysis of genes upregulated in eWAT of wild-type mice in response to HFD challenge and downregulated in *Acod1*^-/-^ counterparts (Fig. 4A and Supplementary Table 3). In particular, *Acod1* loss opposed the generation of a gene expression signature indicative of macrophage accumulation in fat depots, as demonstrated by down-regulation of several macrophage markers (*Adgre1*, *Cd68*, *Lgals3*, *Trem2*, *Mrc1*, *Csf1r*, *Cd80*, *Cd86*, *Cd83*, *Lyz2*, *Cd14* and *Cd180*) and chemokines (*Ccl2*, *Ccl3*, *Ccl4*, *Ccl5, Ccl6*, *Ccl7* and *Ccl9*) promoting macrophage recruitment in tissues (Fig. 4B). In support of such evidence, immunohistochemical detection of macrophage marker F4/80 (*Adgre1*) revealed fewer crown-like structures in eWAT isolated from HFD-fed *Acod1*^*-/-*^ mice compared with wild type counterparts (Fig. 4C, D). Beyond eWAT, *Acod1* ablation decreased mRNA levels of F4/80 (used as a proxy for macrophage accumulation) in iWAT and colon of mice challenged with HFD, compared with their wild-type counterparts (Supplementary Fig. 5D).

Also, expression of genes promoting pro-inflammatory activation of myeloid immune cells upregulated in eWAT of wild type mice in response to HFD, such as *Sting1*, *Lacc1*, *Mif*, *Arl11*, *Zbp1*, several Toll-like receptors (*Tlr1, Tlr2, Tlr6, Tlr7, Tlr13*) and their interactor/adapters (*Tril*, *MyD88* and *Ticam1*), was counteracted by *Acod1* ablation (Fig. 4B). Furthermore, *Acod1* deficiency opposed the transcriptional induction of genes encoding the pro-inflammatory cytokine *Tnf*, its super family members (*Tnfaip8l2*, *Tnfsfm13*, *Tnfsf12*, *Tnfaip2*, *Tnfsf13b*, *Tnfaip8*) and associated receptors (*Tnfrsf11a*, *Tnfrsf1b*, *Tnfrsf13b*, *Tnfrsf21*, *Tnfrsf12a*, *Tnfrsf23*, *Cd40*) promoted by dietary lipid overload in eWAT of wild type mice (Fig. 4B). A transcriptional signature indicative of a decreased inflammatory response was also retrieved in iWAT from HFD-fed *Acod1*^-/-^ mice, compared with wild type counterparts (Supplementary Fig. 5D, E and Supplementary Table 4). In line, *Acod1* ablation decreased *Il1b* mRNA levels in BAT (Fig. 4E) and circulating levels of pro-inflammatory cytokines in plasma (Fig. 4F) of HFD-fed mice. Overall, these data indicate that *Acod1* loss protects mice from metabolic inflammation induced by fat overnutrition.

### Acod1 deficiency ameliorates gut microbiota changes underlying diet-induced obesity

**Fig. 5:**
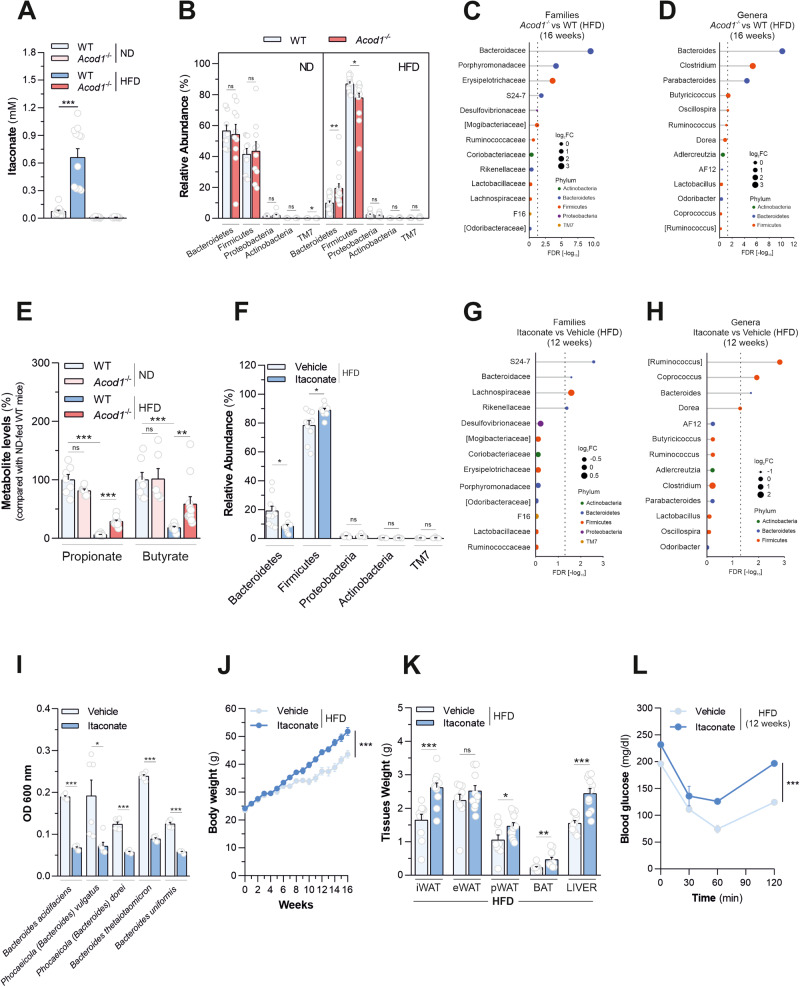
Itaconate production sustains gut microbiota alterations underlying HFD-induced obesity. **A** Fecal itaconate levels of WT and *Acod1*^*-/-*^ mice fed for 16 weeks with ND or HFD. Data as mean±s.e.m. of *n* = 12 mice per group from three experiments independently executed. **B** Fractional analysis of the fecal bacterial composition at the phylum level of mice described in **A**. Data as mean±s.e.m. of *n* = 10 (ND) or *n* = 15 (HFD) mice from three independent experiments. Differential abundance analysis (DESeq2) of the fecal bacterial composition at family (**C**) and genus (**D**) level of WT (*n* = 15) and *Acod1*^*-/-*^ (*n* = 15) mice fed with HFD for 16 weeks from three independent experiments. Dashed line indicates FDR = 0.05. The size of the dots indicates fold change (log2) and the color indicates the phylum. **E** Levels of fecal SCFAs in mice treated as in **A**. Data as mean±s.e.m. of *n* = 7 (ND-fed WT), *n* = 6 (ND-fed *Acod1*^*-/-*^), *n* = 8 (HFD-fed WT), *n* = 11 (HFD-fed *Acod1*^*-/-*^) mice from two experiments independently executed. **F** Fractional analysis of the fecal bacterial composition at the phylum level of HFD-fed WT mice treated with 20 mM itaconate in drinking water for 12 weeks. Data as mean±s.e.m. of *n* = 10 mice per group from two experiments independently executed. Differential abundance analysis (DESeq2) comparing fecal bacterial composition at family (**G**) and genus (**H**) levels of mice described in **F** (*n* = 10 mice per group from two experiments independently executed). Dashed line indicates FDR = 0.05. The size of the dots indicates fold change (log2) and the color indicates the phylum. **I** Effect of itaconate (0.5 mM) treatment on growth (OD 600 nm) of five pure bacteria strains representative of the *Bacteroidaceae* family (*Bacteroides* genus), over 24 h of culture in anaerobic atmosphere, measured spectrophotometrically. Data as mean ± s.e.m. of *n* = 6 samples per condition from two independent experiments. **J** Body weight of HFD-fed WT mice treated with 20 mM itaconate in drinking water for the time indicated. Data as mean ± s.e.m. of *n* = 10 (vehicle) and *n* = 12 (itaconate)-treated mice from two experiments independently executed. **K** Tissue weights from mice escribed in **J**. Data as mean±s.e.m. of *n* = 10 (vehicle) and *n* = 12 (itaconate)-treated mice from two experiments independently executed. **L** ITT in WT mice treated with 20 mM itaconate in drinking water for 12 weeks. Data as mean±s.e.m. of *n* = 5 (vehicle) and *n* = 7 (itaconate)-treated mice from one representative experiment out of two independently executed. In: (**A**), (**B**), (**E**), (**F**), (**I**) and (**K**) *t*-test. In (**J**) and (**L**) two-way ANOVA. *P < 0.05; **P < 0.01; ***P < 0.001; ns not significant.

Intestinal microbiota participates in the development of metabolic disease and its composition can be altered by diet as well as host-derived metabolic signals [34]. Moreover, itaconate biosynthesis is known to regulate interactions between host and microbes [16]. We found that HFD consumption resulted in accumulation of itaconate (~0.66 mM) in stools of HFD-fed wild-type mice, abrogated by *Acod1* ablation (Fig. 5A). Prompted by such observations, we envisioned that resistance of *Acod1*^*-/-*^ mice to diet-induced obesity might be functionally linked to changes in gut microbiota. To address this hypothesis we profiled the bacterial composition of the fecal microbiota isolated from wild type and *Acod1*^*-/-*^ mice fed with either standard chow or HFD, by using 16S ribosomal DNA sequencing. *Bacteroidetes* and *Firmicutes* phyla dominate the intestinal ecosystem in mice and humans and changes in their abundance are associated with metabolic disease [5, 6, 35, 36]. As expected, HFD feeding determined a dramatic decrease in the fecal *Bacteroidetes* to *Firmicutes* ratio (Supplementary Fig. 6A) a dysbiotic signature associated with the obese phenotype [5–7]. However, consistent with obesity amelioration, such ratio in HFD-fed *Acod1*^*-/-*^ mice was ~2.5-fold higher than in wild-type counterparts (Supplementary Fig. 6A). In particular, *Acod1* loss elicited minor, although significant, effects on representation of *Firmicutes* and, predominantly, opposed the decrease in the fecal abundance of *Bacteroidetes* (Fig. 5B and Supplementary Fig. 6B), the major gut microbial phylum associated with metabolic health, depleted in obese mice and humans [5–7]. In line with this, unbiased differential abundance analysis (Fig. 5C), substantiated by relative abundance measurements (Supplementary Fig. 6D, E), revealed that *Bacteroidaceae* (belonging to *Bacteroidetes* phylum) ranked as the most significantly increased bacterial family in stools of *Acod1*-deficient mice fed with HFD, compared to wild type counterparts. Consistent with this, *Bacteroides*, a genus assigned to *Bacteroidaceae* family, scored as the most significantly augmented in stools of *Acod1*-deficient mice, compared to wild-type counterparts challenged with HFD (Fig. 5D and Supplementary Fig. 6F, G). Importantly, members of such taxa have already been extensively associated with protection against obesity and type 2 diabetes in both mice and humans [37–40], in line with the leaner and metabolically healthier phenotype shown by *Acod1*^*-/-*^ mice fed with HFD, compared with wild type controls. On the contrary, no major differences were determined by genotype on representation of such bacterial taxa in mice fed with regular chow (Supplementary Fig. 6C–G). HFD-induced gut dysbiosis is associated with decreased production of the short-chain fatty acids (SCFAs) propionate and butyrate by anaerobic intestinal microbiota, mainly generated by fermentation of dietary fibers by *Bacteroidetes* members, eliciting several beneficial effects on the host [41, 42]. Consistent with fecal microbiota alterations, HFD consumption resulted in decreased SCFA levels in stools of wild-type mice, with *Acod1* loss counteracting such decline (Fig. 5E), pointing toward an amelioration of an obesity-associated intestinal ecosystem in the absence of itaconate. Interestingly, no significant differences were detected in bacterial composition of the small intestine isolated from *Acod1*^*-/-*^ mice fed with HFD compared with wild-type counterparts (Supplementary Fig. 7A–G), consistent with the unchanged *Acod1* gene expression levels detected in such tissue in response to dietary lipid overload (Fig. 1A and Supplementary Fig. 1A). Such results indicate that changes imposed by *Acod1* on the fecal microbiota largely represent a proxy for alterations occurring in the large intestine.

On the basis of such evidence, we hypothesized that further increase of itaconate levels in wild-type mice fed with HFD, beyond the levels endogenously produced, would have mainly promoted the decrease in fecal representation of *Bacteroidetes*, thereby enhancing obesogenic responses to dietary lipid overload. To test this, we tied the impact of *ad libitum* oral administration of high dose of itaconate (20 mM) in wild-type mice - resulting in ~5-fold increase of fecal itaconate levels compared with vehicle-treated counterparts (Supplementary Fig. 8A) - on fecal microbiota composition with host responses induced by HFD consumption. Consistent with the increased metabolite abundance, and specularly to the effects imposed by *Acod1* loss on fecal microbiota, itaconate administration halved the fractional abundance of *Bacteroidetes* in stools of mice fed with HFD for 12 weeks (Fig. 5F and Supplementary Fig. 8B) and, as a consequence, decreased their *Bacteroidetes* to *Firmicutes* ratio (Supplementary Fig. 8C). At lower taxonomical level, such alteration was mainly coupled with a significantly reduced representation of *Bacteroidaceae* (Fig. 5G and Supplementary Fig. 8D, E) and *Bacteroides* (Fig. 5H and Supplementary Fig. 8F, G), the two bacterial taxa, associated with metabolic health, most significantly increased in stools of HFD-fed *Acod1*^-/-^ mice, with respect to wild type counterparts (Fig. 5C, D). It is worth noting that both the impact of *Acod1* loss and oral itaconate administration on fecal microbiota composition were reasonably attributable to a direct action on intestinal bacteria, as demonstrated by the dose-dependent decrease of *Bacteroidetes* levels (Supplementary Fig. 8H), *Bacteroidetes* to *Firmicutes* ratio (Supplementary Fig. 8I), *Bacteroidaceae* (Supplementary Fig. 8J), and *Bacteroides* (Supplementary Fig. 8K) abundances measured in a fecal suspension deriving from pooled stools of HFD-fed wild-type mice, following incubation in vitro with itaconate (0.5–20 mM) in anaerobic atmosphere, compared with vehicle-treated controls. In line with these data, administration of near-physiologically relevant concentration of itaconate (0.5 mM) resulted in inhibition of in vitro growth of five pure bacterial strains representative of the *Bacteroidaceae* family (*Bacteroides* genus) – *Bacteroides acidifaciens, Phocaeicola (Bacteroides) vulgatus, Phocaeicola (Bacteroides) dorei, Bacteroides thetaiotaomicron* and *Bacteroides uniformis*) [39, 40, 43–47] – already reported to have beneficial roles against metabolic disease (Fig. 5I). Consistent with a more pronounced obesity-associated microbial signature, itaconate-treated mice showed enhanced obesogenic responses following HFD-consumption, as demonstrated by higher body weight gain (Fig. 5J), increased weight of several adipose tissues and liver (Fig. 5K), augmented hepatic steatosis (Supplementary Fig. 8L), decreased hepatic mRNA levels of *Nags*, *Cps1* and *Ass1* urea cycle genes (Supplementary Fig. 8M), increased F4/80 (*Adgre1*) mRNA levels (indicative of macrophage accumulation) in eWAT and iWAT (Supplementary Fig. 8N) as well as lower insulin sensitivity (Fig. 5L and Supplementary Fig. 8O) with no changes in food intake (Supplementary Fig. 8P), with respect to vehicle-treated counterparts. In all, such data indicate a pivotal role for itaconate, either endogenously produced or exogenously administered, in supporting gut microbiota changes underlying diet-induced metabolic disease.

### Itaconate biosynthesis promotes metabolic disease by sustaining gut microbiota alterations driving meta-inflammation

**Fig. 6:**
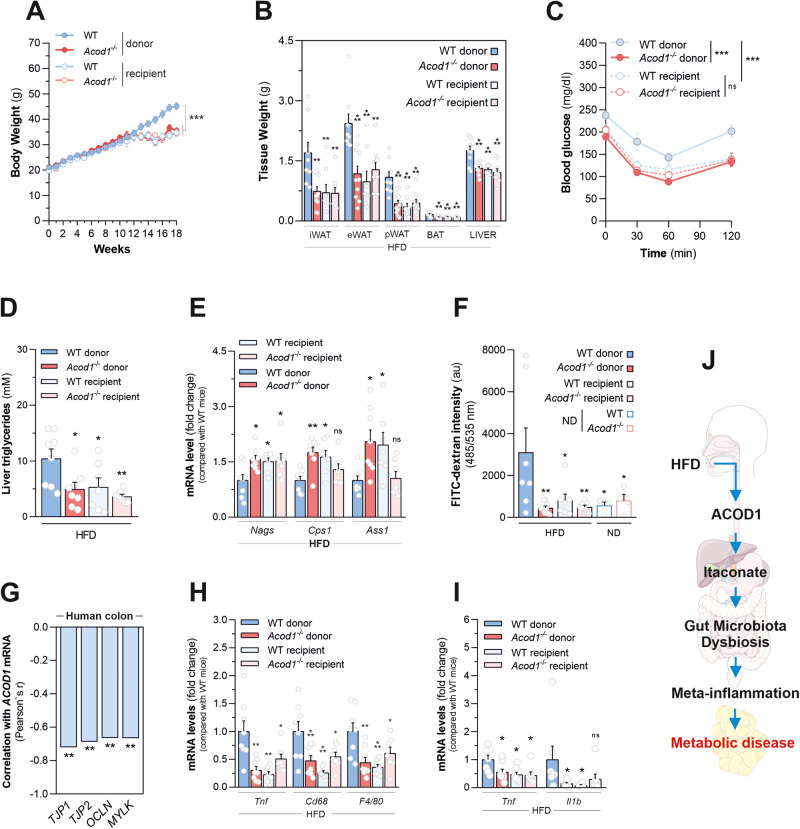
Fecal microbiota of *Acod1*-deficient mice offers transferable protection from metabolic disease by preventing meta-inflammation. Body (**A**) and tissue (**B**) weights of HFD-fed WT and *Acod1*^*-/-*^ mice receiving either PBS (donor) or a fecal suspension from reciprocal genotypes (recipient). Data as mean ± s.e.m from *n* = 9 (WT donor), *n* = 11 (A*cod1*^*-/-*^ donor), *n* = 8 (WT recipient) and *n* = 10 *Acod1*^*-/-*^ recipient mice from two experiments independently executed. **C** ITT from mice treated as in **A**. Data as mean±s.e.m of *n* = 9 (WT donors), *n* = 11 (A*cod1*^*-/-*^ donors), *n* = 8 (WT recipient) and *n* = 9 *Acod1*^*-/-*^ recipient mice from two experiments independently executed. **D** Liver triglycerides and (**E**) qPCR analysis of indicated hepatic genes of mice treated as in **A**. Data as mean±s.e.m from *n* = 8 (donors) and *n* = 7 (recipient) mice from two independent experiments. **F** Plasma FITC-dextran levels in ND-fed mice and mice treated as in **A**. Data as mean±s.e.m from *n* = 4 (ND), *n* = 7 (WT donor), *n* = 9 (*Acod1*^*-/-*^ donor), *n* = 7 (WT recipient), and *n* = 8 (*Acod1*^*-/-*^ recipient) mice from two independent experiments. **G** Correlation analysis between mRNA of *ACOD1* and the indicated genes in colon specimens of human obese (*n* = 16) subjects (GSE158237). **H** qPCR analysis of indicated genes in eWAT of mice treated as in **A**. Data as mean±s.e.m from *n* = 8 (donors) and *n* = 7 (recipient) mice from two independent experiments. **I** qPCR analysis of indicated genes in BAT of mice treated as in **A**. Data as mean±s.e.m from *n* = 7 (WT donor), *n* = 9 (*Acod1*^*-/-*^ donor) and *n* = 7 (recipient) mice from two independent experiments. **J** Diagram summarizing ACOD1-driven metabolic disease sequelae. In (**A**), (**C**) two-way ANOVA; In (**B**), (**D**–**F**), (**H**, **I**) one-way ANOVA with Dunnett’s multiple comparisons test compared with WT donor; *P < 0.05; **P < 0.01; ***P < 0.001; ns not significant.

Data shown so far prompted us to determine the extent to which gut microbiota alterations, determined by genetic disruption of itaconate biosynthesis, account for resistance to diet-induced obesity and its complications. For this aim, we measured responses to overnutrition in both wild type and *Acod1*^*-/-*^ (recipient) mice after prolonged oral transfer of feces from their reciprocal genotype (donor) counterparts, all fed with HFD. No major differences in the fecal composition at any taxonomic level were imposed by genotype in the groups used before the fecal transplantation (Supplementary Fig. 9A–H). On the contrary, a marked increase in fecal representation of *Bacteroidetes* was detected in stools of wild type recipients with respect to wild-type donor counterparts, at the end of the experiment (Supplementary Fig. 10A, B). In line with phylum-level data, the differential abundance matrixes (Supplementary Fig. 10C, F) and fractional analyses (Supplementary Fig. 10E, G, H), carried out at family and genus level, show that the transplant of stools from *Acod1*^*-/-*^ donors increased the levels of *Bacteroidaceae* and *Bacteroides* - bacterial taxa associated with protection from metabolic disease - into wild type recipients compared with wild type donor mice. Consistent with a less pronounced obesity-associated microbial signature, wild-type mice receiving fecal microbiota of *Acod1*^*-/-*^ littermates gained less weight (Fig. 6A), showed lower fat depots and liver weights (Fig. 6B) as well as increased insulin sensitivity (Fig. 6C and Supplementary Fig. 11A), compared with wild type donors. In line with changes in liver weight, the transplant of *Acod1*^*-/-*^ fecal microbiota in wild-type mice also decreased liver steatosis (Fig. 6D) and increased hepatic mRNA levels of *Nags*, *Cps1* and *Ass1* urea cycle genes (Fig. 6E). Contrarily, no major differences were observed in the fecal microbiota composition of *Acod1*^*-/-*^ recipients compared with *Acod1*^*-/-*^ donor mice (Supplementary Fig. 10A–H). These results might be explained by the intrinsic resistance of the *Acod1*^*-/-*^ hosts to colonization of microbiota of wild-type mice – despite a marked and comparable depletion of fecal bacterial content was obtained in both genotypes in response to antibiotics treatment preceding fecal administration (Supplementary Fig. 9I) - and/or the insufficient residual levels of itaconate, derived from the amount of wild type stools gavaged (Supplementary Fig. 10I), in affecting microbiota composition of *Acod1*^*-/-*^ recipients. In agreement with the similar fecal metagenomic composition, no major differences in body weight gain (Fig. 6A), fat depot weight (Fig. 6B), responsiveness to insulin (Fig. 6C and Supplementary Fig. 11A) as well hepatic lipid accumulation (Fig. 6D) and urea cycle gene expression (Fig. 6E) were observed in *Acod1*^*-/-*^ mice receiving stools from wild type counterparts, with respect to *Acod1*^*-/-*^ controls. Overall, these data indicate a dominant protective effect of fecal microbiota from *Acod1*^*-/-*^ mice against diet-induced obesity. HFD-induced gut dysbiosis might result in increased intestinal permeability, contributing to the generation of meta-inflammation in fat depots. Consistent with obesity resistance, *Acod1* loss lowered FITC-dextran levels increasing in serum of wild-type mice following oral dye administration, in response to HFD consumption (Fig. 6F), indicating preserved gut barrier function. In line with this, *ACOD1* mRNA levels were found significantly negatively correlated with the expression of genes forming and preserving the integrity of tight junction paracellular intestinal permeability barrier (*TJP1*, *TJP2, OCLN* and *MYLK*) (Fig. 6G and Supplementary Table 1), hypo-expressed in colon specimens of obese subjects with respect to healthy controls (Supplementary Fig. 11B and Supplementary Table 1), thereby strengthening the role for ACOD1 in derailing gut barrier function also in humans. Importantly, the transplant of *Acod1*^*-/-*^ stools in wild-type mice decreased gut leakiness (Fig. 6F) and, as a consequence, down-regulated the inflammatory transcriptional signature in adipose tissues (Fig. 6H, I) as well as increased the expression of several energy expenditure-promoting genes in BAT (Supplementary Fig. 11C). Taken together, these data demonstrated that abrogation of itaconate biosynthesis offers an amelioration against diet-induced obesity by maintaining a healthy gut microbiota limiting meta-inflammation and its associated outcomes (Fig. 6J).

## Discussion

Obesity imposes a heavy burden on the health care system by increasing the risk of severe chronic diseases [1]. Here, we demonstrate that inhibition of itaconate biosynthesis, achieved by genetic disruption of *Acod1* gene protects mice against diet-induced obesity by preserving a healthy gut microbiota opposing meta-inflammation and associated outcomes. *Acod1* loss decreases body weight gain, white adipose tissue accumulation, and adipocyte hypertrophy promoted by fat overnutrition in mice. Also, *Acod1* deficiency protects mice from alterations in glucose homeostasis induced by HFD consumption. Importantly, such effects were abrogated by oral administration of low dose of itaconate (1 mM) to HFD-fed *Acod1*^*-/-*^ mice, indicating an instrumental role for itaconate production in development of metabolic disease. Consistent with resistance to diet-induced obesity, *Acod1* deficiency decreases liver steatosis, dampening the hepatic accumulation of free fatty acids and associated diacylglycerol species, known to have causal roles in lipid-induced hepatic insulin resistance [48]. Furthermore, by proteomics and analytical chemistry-integrated approaches we demonstrated that *Acod1* ablation opposes the urea cycle dysfunction promoted by liver steatosis, known to play an instrumental role in the progression of NAFLD [25, 26]. Interestingly, a recently published study demonstrated that itaconate production by liver macrophages of mice fed with Western diet, combined with oral sucrose administration, opposes NAFLD [49]. It is worth noting that such cholesterol-rich dietary regimen is known to induce severe steatohepatitis and fibrosis in mice [50–52], contrarily to consumption of HFD as used in our study, suggesting that the role of ACOD1 in metabolic liver disease might be diet-dependent. Also, during the preparation of this manuscript, a report investigating the role of *Acod1* in obesity has been released [53]. Contrary to our findings, Frieler and co-workers show that *Acod1* ablation in mice elicits no impact on body weight gain and worsens dysfunction of glucose homeostasis induced by HFD consumption for a shorter time period (12 weeks), compared with our dietary regimen. Moreover, insulin resistance in *Acod1*^*−/−*^ mice fed with normal diet for 12 months was reported. Although exploring aging-dependent metabolic effects resulting from *Acod1* deficiency are beyond the aim of the present work, our results demonstrate that *Acod1* plays no major roles in regulating body weight, fat depots, glycemic control, liver steatosis and obesity-associated gut microbiota composition in mice fed with normal chow for ~5 months. Although both studies were performed by comparing the effects of genotype among littermates, differences in genetic background of mice, vivaria as well as housing conditions might explain the discrepancies between their data and our results. In this regard, our experiments were performed with mice separated in cages by genotype (individually caged), in order to avoid spontaneous transfer of microbiota between strains by coprophagy, which could mask or confound the responses imposed by *Acod1* loss to diet administration. Contrarily, no indication of caging conditions were reported in that study.

By using whole-body indirect calorimetry, we demonstrated that *Acod1* deficiency offers resistance to diet-induced obesity by stimulating energy expenditure. Importantly, energy expenditure and glucose homeostasis are altered by the emergence of a chronic low-grade inflammatory state associated with overnutrition. Here, we report that *Acod1* deficiency decreased macrophage accumulation in adipose tissue, suppressed the pro-inflammatory transcriptional profile of fat depots and dampened the levels of circulating pro-inflammatory mediators in HFD-challenged mice. Collectively, such data demonstrate that *Acod1* has an instrumental role in sustaining meta-inflammatory responses to dietary lipid overload in mice.

Growing evidence points towards an instrumental role of ACOD1 and itaconate in promoting bacterial replication, sustaining pro-inflammatory cytokine production, and lethal innate immune responses in mouse models of experimental endotoxemia and microbial sepsis [16, 21, 54]. Therefore, we envisioned that the effects of *Acod1* ablation in counteracting meta-inflammation and obesity sequelae might be secondary to changes in gut microbiota, contributing to regulate inflammatory responses and metabolic health in the host. Profiling of mouse fecal microbiota supported our hypothesis, unveiling a role for *Acod1* in promoting the decrease in the ratio between *Bacteroidetes* and *Firmicutes*, the two major bacterial phyla of the intestinal ecosystem, induced by HFD consumption. Such dysbiotic signature is frequently associated with obesity in both mice and humans [5, 6, 35, 36] and is restored after anti-obesogenic interventions, with *Bacteroidetes* proportion positively correlating with body fat loss [6, 55]. In particular, *Acod1* loss elicited minor effects on representation of *Firmicutes* and, predominantly, opposed the decrease in the fecal abundance of *Bacteroidetes*, the major gut microbial phylum associated with metabolic health, depleted in obese mice and humans [5–7]. In more detail, unbiased differential abundance analyses identified *Bacteroidaceae* and *Bacteroides* as the most significantly increased bacterial family and genus, respectively, in stools of *Acod1*-deficient mice challenged with HFD compared to wild-type counterparts. Importantly, members of such taxa have already been extensively associated with protection from obesity and type 2 diabetes [37–40, 43–47], in line with the amelioration of metabolic disease offered by *Acod1* loss in mice. Interestingly, no significant alterations were detected in bacterial composition of the small intestines isolated from *Acod1*^*-/-*^ mice fed with HFD, compared with wild-type counterparts. Such results indicate that changes imposed by *Acod1* loss on the fecal microbiota largely represent a proxy for alterations occurring in the large intestine. Several studies point to *Bacteroidetes* as the largest SCFA producers in mouse and human gut [56–58]. These metabolites elicit anti-obesogenic effects by promoting energy expenditure in fat depots, ameliorating whole-body glucose homeostasis, and opposing the production of pro-inflammatory mediators by immune cells [8]. SCFAs also sustain gut barrier function. Increased intestinal permeability, resulting from diet-induced intestinal dysbiosis, favors the translocation of micro-organisms and microbial-associated molecular patterns through the gut epithelium, thereby promoting obesity-associated inflammatory responses in the host [8, 9, 59]. In line with a sustained fecal *Bacteroidetes* abundance and protection from diet-induced meta-inflammation, *Acod1* loss opposed both the decline of fecal SCFA levels and the alteration of gut barrier function, induced by fat overnutrition in mice. Importantly, the amelioration of the mouse intestinal ecosystem, altered by HFD consumption, is not just the result of the protection from diet-induced obesity achieved by *Acod1* deficiency, but contributes instrumentally to generate it. Such a conclusion derives from the evidence of dominant protective effects against adipose tissue accumulation, meta-inflammation, and obesity-associated metabolic dysfunctions offered by the transfer of fecal microbiota from HFD-fed *Acod1*^*-/-*^ mice into wild-type counterparts. Furthermore, it is noteworthy that major changes in fecal microbiota composition induced by the genetic disruption of itaconate biosynthesis were specular to those induced by oral administration of a supraphysiological dose (20 mM) of itaconate to mice fed with HFD. In detail, the increase of fecal itaconate abundance in wild-type mice, beyond the levels endogenously produced following dietary lipid consumption, mainly halved the relative proportion of *Bacteroidetes, Bacteroidaceae,* and *Bacteroides* taxa in fecal microbiota of HFD-fed mice. Consistent with such more pronounced obesity-associated microbial signature, supraphysiological itaconate supplementation enhanced overall metabolic responses (i.e., body weight gain, fat depot accumulation, liver steatosis, and insulin resistance) of wild-type mice to HFD. Taken together, such results point towards a key role for itaconate in supporting gut microbiota changes underlying diet-induced obesity and associated metabolic dysfunctions.

Beyond diet, host-derived metabolic signals, produced either in peripheral tissues or locally in the intestine, can shape gut microbiota composition and function [11, 60, 61]. We found that HFD consumption promotes up-regulation of *Acod1* gene expression and itaconate production in colon of mice starting from twelve weeks of HFD administration. Importantly, such timing paralleled the divergence in body weight gain between wild type and *Acod1*^*-/-*^ mice challenged with HFD, thus substantiating an instrumental role for colonic *Acod1* expression in the metabolic responses of mice to prolonged fat overnutrition. In line with this, analyses of a publically available transcriptomic dataset of human subjects with obesity retrieved colonic *ACOD1* gene expression tied to obesity, altered glycemic control, and reduced expression of genes preserving gut barrier function. Collectively, such data point towards gut microbiota responsiveness to the induction of itaconate biosynthesis occurring in the large intestine. Investigations are currently in progress to determine how itaconate supports changes in gut microbiota underlying diet-induced obesity. We found that host-derived itaconate accumulates in stools of mice following HFD consumption. Furthermore, we provided evidence for a direct effect of itaconate, used at near-physiologically relevant concentration, in decreasing in vitro growth of pure bacterial strains representative of the *Bacteroidaceae* family (*Bacteroides* genus), previously demonstrated to elicit beneficial roles against metabolic disease. Therefore, it is possible to envision a direct paracrine role for such a metabolite, secreted into the intestinal lumen, in altering equilibria among commensal bacteria in response to dietary changes. However, as the human ACOD1 is less active than murine counterpart [62, 63] at this stage it is not clear whether any increase in itaconate biosynthesis in human colon might be high enough to be pathophysiologically relevant.

In conclusion, by unveiling an unrecognized impact of *Acod1* loss on diet-induced gut microbiota alterations, this study has uncovered a novel role for itaconate biosynthesis in obesity and major associated inflammatory outcomes induced by fat overnutrition, paving the way for the development and utilization of synthetic small-molecule inhibitors targeting ACOD1 activity for treatment of metabolic disease.

## Methods

### Mice and treatments

Male C57BL/6 *Acod1*^*−/−*^ (C57BL/6N-Acod1^em1(IMPC)J^/J) were bought from the Jackson Laboratory and bred in house. Mouse genotype was determined by amplification of DNA from *tail* biopsies by using Phire Tissue Direct PCR Master Mix (F170S, Thermo Scientific™) and allele-specific primers indicated by Jackson Laboratory (Supplementary Table 5). In experiments comparing wild type and *Acod1*^*-/-*^ genotypes, experimental littermates were used and generated breeding *Acod1*^*+/-*^ mice together. In experiments were only wild type littermates were used, they were generated by breeding wild-type mice together. At weaning, all mice were separated according to the genotype and housed together in (at least two) different cages (multi-housed) until five weeks of age. Then, starting from five weeks of age, mice were individually caged, randomized, blindly allocated to experiment groups and fed with either a standard chow or high-fat diet (HFD) containing 60% of calories from fat (E15742-347, Charles River) for 16 weeks (if not otherwise stated throughout the main text or in each figure caption), with ad libitum access to water and food. For itaconate treatments, mice received either 1 mM or 20 mM neutralized itaconate solution in drinking water ad libitum during HFD feeding, as indicated in the main text/figure captions. In all HFD-feeding experiments, body weight of mice was measured weekly. Tissues were collected at the end of each experiment for post-mortem analyses. The investigators were not blinded during experiments and outcome assessment.

### Human datasets

The microarray GSE158237 [64] dataset integrates RMA normalized mRNA expression values from colonic biopsies and clinical data of human subjects with different degrees of body mass index (BMI). Healthy (BMI < 25 kg/m^2^, *n* = 13) and obese (BMI > 30 kg/m^2^, *n* = 16) subjects were included in the analysis. To correlate colonic *ACOD1* mRNA levels and tolerance to glucose overload, relative changes in plasma glucose levels measured 2 h after bolus glucose administration were compared with their baseline levels. The RNAseq dataset GSE130970 [65] contains transcriptomic profiles of 78 distinct human liver biopsies. Of these, 6 are histologically normal and 72 cover the full spectrum of nonalcoholic fatty liver disease (NAFLD) (assessed by NIDDK NASH CRN criteria [66]. In both studies, data were tested for normality [67–80].

### Ethical approval of animal studies

All mice used in this study were bred and housed in individual ventilated cages in a barrier facility proactive in environmental enrichment under specific pathogen-free conditions in line with European Union regulations. All experimental animal procedures were approved by the Institutional Animal Committee of San Raffaele Scientific Institute.

### Statistics and reproducibility

For each experiment, sample size was chosen on the basis of similar experimental approaches reported in the literature. If not otherwise stated, data are expressed as mean ± s.e.m. and details about group sizes (n) and how many times each experiment was independently repeated is provided in each figure caption. When two groups were compared, statistical significance was evaluated by an unpaired, two-tailed Student’s t-test (referred to as t-test) and applying Welch’s correction when variances between groups were significantly different. For multiple group analyses one-way ANOVA followed by Dunnett’s multiple comparisons test or two-way ANOVA were performed. Mann-Whitney *U*-test (referred to as Mann–Whitney test) was applied in Supplementary Fig. 1D, E, as data were not normally distributed. Pearson’s correlation was applied to determine the relationship between *ACOD1* mRNA expression levels and alterations in plasma glucose or alterations in expression of genes related to intestinal permeability. All statistical analyses were performed using GraphPad Prism version 8.

### Supplementary information

Supplemental Information

Supplementary Figure 1

Supplementary Figure 2

Supplementary Figure 3

Supplementary Figure 4

Supplementary Figure 5

Supplementary Figure 6

Supplementary Figure 7

Supplementary Figure 8

Supplementary Figure 9

Supplementary Figure 10

Supplementary Figure 11

Supplementary Table 1

Supplementary Table 2

Supplementary Table 3

Supplementary Table 4

Supplementary Table 5

checklist

## Data Availability

Proteomics and RNAseq data are available at ProteoSAFe/MassIVE (MSV000090276) and GEO (GSE213632), respectively. All other data that support the findings in this study are stored at the IRCCS San Raffaele Scientific Institute and are available from the corresponding author upon reasonable request.
