# Supplementary material for: ACOD1 deficiency offers protection in a mouse model of diet-induced obesity by maintaining a healthy gut microbiota
Source: Cell Death Dis. 2024 Feb 1;15(2):105. doi: 10.1038/s41419-024-06483-2 (PMC10834593; doi:10.1038/s41419-024-06483-2)
Supplement: Supplementary file 1 — Supplemental Information [file 41419_2024_6483_MOESM1_ESM.docx]

**Supplementary Information**

**ACOD1 deficiency offers protection in a mouse model of diet-induced obesity by maintaining a healthy gut microbiota**

Eberhart and Stanley et al.

**Supplementary Figure 1. Genetic Acod1 deficiency protects mice from diet-induced obesity.** (**A**) qPCR analysis of *Acod1* mRNA levels in the indicated tissues from WT and *Acod1^-/-^* mice fed with ND or HFD for the time indicated. Data as mean±sem of n=4-6 mice per group from one experiment. (**B**) Itaconate levels in colon from the indicated mice fed with either ND or HFD for 12 weeks. Data as mean±sem of n=5 (ND-fed) and n=4 (HFD-fed) mice from one experiment. (**C**) qPCR analysis of F4/80 (*Adgre1*) mRNA levels in the indicated tissues from WT mice fed with ND or HFD for the time indicated. Data as mean±sem of n=4-7 mice per group from one experiment. (**D**) ACOD1 mRNA levels (GSE130970) in liver of NAFLD patients categorized according to disease (NAFL) score. (**E**) *ACOD1* mRNA levels (GSE130970) in liver of NAFLD patients categorized according to hepatic steatosis grade. (**F**) Weights (g) of the indicated tissues isolated from WT and *Acod1^-/-^* mice after 16 weeks on HFD. Data as mean±s.e.m of n=7 (HFD-fed WT), n=6 (HFD-fed *Acod1^-/-^*) mice from one representative experiment out of three independently executed. (**G**) Quantification of adipocyte size in images described in Fig. 1H. Data as mean±s.e.m. (n = 5 mice per group, average of 5 images each). (**H**) Quantification of BAT whitening in images described in Fig. 1I. Data as mean±s.e.m. (n = 4 mice per group, average of 3 images each); FOV, field of vision. (**I**) Area under the curves (AUC) of GTT described in Fig. 1L. ND (n=6), HFD (n=7). Data as mean±sem of n=6 (ND) and n=7 (HFD) from one representative experiment out of two independently executed. (**J**) Area under the curves of ITT described in Fig. 1M. Data as mean±sem of n=5 (ND) and n=7 (HFD) from one representative experiment out of two independently executed. (**K**) Levels of metabolic hormones measured in plasma of WT and *Acod1^-/-^* mice after 16 weeks on ND or HFD**.** Data as mean±s.e.m of n=4 (ND-fed), n=7 (HFD-fed) mice from one experiment. In (A, B, C, F, G, H, K): t-test; (D-E): Mann-Whitney test; (I, J) one-way ANOVA followed by Dunnett's multiple comparisons test. **P*<0.05; ***P*<0.01; ****P*<0.001; ns: not significant.

**Supplementary Figure 2. Itaconate rescues the effects of *Acod1* ablation on metabolic responses to HFD.** (**A**) Determination of itaconate levels in colon of HFD-fed mice in response to treatment with 1mM itaconate in drinking water for 16 weeks. Data as mean±s.e.m of n=5 (WT), n=7 (*Acod1^-/-^*) and n=5 (*Acod1^-/-^* + Itac.) mice from one experiment. (**B**) Effect of treatment of 1mM itaconate in drinking water on body weights of *Acod1^-/-^* mice fed with HFD for 16 weeks. Data as mean±s.e.m of n=5 (WT), n=7 (*Acod1^-/-^*) and n=5 (*Acod1^-/-^* + Itac.) mice from one experiment. (**C**) Tissue weights of mice described in (L). Data as mean±s.e.m of n=5 (WT), n=7 (*Acod1^-/-^*) and n=5 (*Acod1^-/-^* + Itac.) mice from one experiment. (**D**) ITT (left) and AUC of ITT (right) in mice described in (B). Data as mean±s.e.m of n=5 (WT), n=7 (*Acod1^-/-^*) and n=5 (*Acod1^-/-^* + Itac.) mice from one experiment. In (A, C and D-right panel): one-way ANOVA followed by Dunnett's multiple comparisons test. In (B and D-left panel): two-way Anova. **P*<0.05; ***P*<0.01; ****P*<0.001; ns: not significant (t-test).

**Supplementary Figure 3. *Acod1* ablation opposes liver steatosis and associated metabolic dysfunction induced by dietary lipid overload.** (**A**) Principal component analysis (PCA) comparing liver proteome of WT and *Acod1^-/-^* mice fed with either ND or HFD for 16 weeks*.* Data derive from n=5 ND-fed (WT), n=8 (ND-fed *Acod1^-/-^*) and n=6 (HFD-fed) mice from two independent experiments. (**B**) Pearson`s correlation analysis between changes in the abundance of liver proteins imposed by dietary lipid overload in WT mice (HFD vs ND [WT]) and those determined by *Acod1* loss in mice on HFD (*Acod1^-/-^* vs WT [HFD]. Statistically significantly altered proteins (FRD < 0.1) are illustrated in blue, not statistically significantly altered proteins in white. FC: fold change. Data derive from n=5 ND-fed (WT), n=8 (ND-fed *Acod1^-/-^*) and n=6 (HFD-fed) mice from two independent experiments. (**C**) qPCR analysis of mRNA levels of the indicated genes in livers of WT and *Acod1^-/-^* mice fed with either ND or HFD for 16 weeks*.* Data as mean±s.e.m of n=12 (ND-fed WT), n=16 (ND-fed *Acod1^-/-^*) and n=15 (HFD-fed) mice from three independent experiments; **P*<0.05; ***P*<0.01; ****P*<0.001 (one-way ANOVA followed by Dunnett's multiple comparisons test, compared with HFD-fed WT).

**Supplementary Figure 4. *Acod1* loss improves metabolic activity of HFD-fed mice.**

(**A**) Measurement of food intake of WT and *Acod1^-/-^* mice during light and dark hours after 16 weeks on HFD; n=6 (WT), n=7 (*Acod1^-/-^*) mice. Data as mean±s.e.m from one representative experiment out of two independently executed. (**B**) Bomb-calorimetry-mediated determination of energy content in stools of WT (n=7) and *Acod1^-/-^* (n=5) mice on HFD for 16 weeks. Data as mean±s.e.m from one representative experiment. (**C**) Neutral lipid content (oil red staining) of adipocytes isolated from iWAT of WT and *Acod1^-/-^* mice, on day 0 and day 5 of differentiation *in vitro*; n= 4 replicates per group. Data as mean±s.e.m from one representative experiment out of three independently executed. (**D**) qPCR analysis of *Pparg* and *Cebpa* in WT and *Acod1^-/-^* adipocytes described in (C); n= 3-6 replicates per group. Data as mean±s.e.m from one representative experiment out of three independently executed. (**E**) Respiratory exchange rate (RER) of WT and *Acod1^-/-^* mice described in (A); n=6 (WT), n=7 (*Acod1^-/-^*) mice. Data as mean±s.e.m from one representative experiment out of two independently executed. (**F**) Plasma leptin levels in WT and *Acod1^-/-^*; n=3 (ND-fed), n=7 (HFD-fed) mice. Data as mean±s.e.m from one representative experiment out of two independently executed. (**G**) Total locomotor activity (cm) of mice described in (A); n=6 (WT), n=7 (*Acod1^-/-^*) mice. Data as mean±s.e.m from one representative experiment out of two independently executed. (**H**) qPCR analysis of *Pgc-1α* (*Ppargc1a*) mRNA levels in eWAT of WT and *Acod1^-/-^* mice fed with either ND or HFD for 16 weeks; n=4 (ND-fed), n=7 (HFD-fed) mice. Data as mean±s.e.m from one representative experiment out of two independently executed. (**I**) Quantification of UCP1 levels in images described in Fig. 3H. Data as mean±s.e.m. (n = 3 mice per group, average of 3 images each). ***P*<0.01 (one-way Anova followed by Dunnett`s T3 multiple comparison test compared with HFD-fed WT mice). (**J**) qPCR analysis of the indicated genes in quadriceps of WT and *Acod1^-/-^* mice on HFD for 16 weeks; n=8 mice per group. Data as mean±s.e.m from one representative experiment out of two independently executed. (**K**) qPCR analysis of the indicated genes in tibialis anterior of WT and *Acod1^-/-^* mice on HFD for 16 weeks; n=7 (WT), n=8 (*Acod1^-/-^*) mice. Data as mean±s.e.m from one representative experiment out of two independently executed. In (A-H, J, K): **P*<0.05 (t-test); ns: not significant.

**Supplementary Figure 5. *Acod1* loss decreases diet-induced obesity-associated meta-inflammation**. (**A**) qPCR analysis of *Acod1* mRNA levels in resting and LPS-activated bone marrow-derived macrophages (BMDMs). Data as mean±s.e.m of n=2 technical replicates per condition from one experiment out of two independently executed. (**B**) ELISA-mediated determination of IL-6 secreted in media of BMDMs treated as in (A). Data as mean±s.e.m of n=6 technical replicates per condition from one experiment out of two independently executed. ****P*<0.001 (t-test). (**C**) ELISA-mediated determination of IL-1β secreted in media of BMDMs treated as in (A) incubated with 2.5 mM ATP for additional 40 min for inflammasome activation. Data as mean±s.e.m of n=6 technical replicates per condition from one experiment out of two independently executed. ****P*<0.001 (t-test). (**D**) qPCR analysis of F4/80 (*Adgre1*) mRNA levels in the indicated tissues from *wild type* and *Acod1^-/-^* mice fed with either ND or HFD for 16 weeks. Data as mean±s.e.m of n= 3-7 mice per group from one representative experiment. ***P*<0.01; **P*<0.05 (one-way Anova followed by Dunnett`s T3 multiple comparison test compared with HFD-fed WT mice); ns: not significant. (**E**) Venn diagram (left) and gene ontology (biological processes)-based functional enrichment analysis (right) of statistically significant (FDR<0.1) up-regulated genes in iWAT of WT mice in response to HFD (HFD vs ND [WT]) and rescued by *Acod1* loss (*Acod1^-/-^* vs WT [HFD]). Data derive from n=3 (ND-fed WT), n=5 (HFD-fed WT) and n=3 (HFD-fed *Acod1^-/-^*) mice from one experiment. (**F**) Heatmap depicting the mRNA levels of selected genes defining macrophage markers, inflammatory cytokines and receptors, regulators of innate immunity, TLR signaling and TNF signaling upregulated in response to HFD (HFDvsND in WT, FDR<0.1) and rescued by *Acod1* loss (*Acod1^-/-^* vsWT in HFD, FDR<0.1) in iWAT. Data derive from mice described in (E): n=3 (ND-fed WT), n=5 (HFD-fed WT) and n=3 (HFD-fed *Acod1^-/-^*) mice from one experiment.

**Supplementary Figure 6. Itaconate production sustains gut microbiota alterations underlying HFD-induced obesity.** (**A**) *Bacteroidetes* to *Firmicutes* ratio in the fecal microbiota of WT and *Acod1^-/-^* mice fed for 16 weeks with ND or HFD. Data as mean±s.e.m. of n=10 (ND) or n=15 (HFD) mice from three experiments independently executed. (**B**) Relative changes in the abundance of bacterial phyla in stools derived from mice described in (A). Data as mean±s.e.m. of n=10 (ND) or n=15 (HFD) mice from three experiments independently executed. (**C**) Differential abundance analysis (DESeq2) of the fecal bacterial composition at family (left) and genus (right) level of WT (n=10) and *Acod1^-/-^* (n=10) mice fed with ND for 16 weeks from three experiments independently executed. Dashed line indicates FDR = 0.05. The size of the dots indicates fold change (log2) and the color indicates the phylum. Fractional analysis (**D**) and relative changes (**E**) of the fecal bacterial composition at the family level of mice described in (A). Data as mean±s.e.m. of n=10 (ND) or n=15 (HFD) mice from three experiments independently executed. Fractional analysis (**F**) and relative changes (**G**) of the fecal bacterial composition at the genus level of mice described in (A). Data as mean±s.e.m. of n=10 (ND) or n=15 (HFD) mice from three experiments independently executed. In (A), (B), (D-G): **P*<0.05; ***P*<0.01 (t-test); ns: not significant.

**Supplementary Figure 7. Effects of *Acod1* loss on the composition of the small intestine microbiota of HFD-fed mice.** Fractional analysis (**A**) and relative changes (**B**) of bacterial composition at the phylum level of the small intestine of WT and *Acod1^-/-^* mice fed for 16 weeks with HFD. Data as mean±s.e.m. of n=10 (WT) or n=12 (*Acod1^-/-^*) mice from three experiments independently executed. (**C**) Differential abundance analysis (DESeq2) of the bacterial composition at family (left) and genus (right) level of WT (n=10) and *Acod1^-/-^* (n=12) mice fed with HFD for 16 weeks, from two experiments independently executed. Dashed line indicates FDR = 0.05. The size of the dots indicates fold change (log2) and the color indicates the phylum. Fractional analysis (**D**) and relative changes (**E**) of bacterial composition at the family level of the small intestine of mice described in (A). Data as mean±s.e.m. of n=10 (WT) or n=12 (*Acod1^-/-^*) mice from three experiments independently executed. Fractional analysis (**F**) and relative changes (**G**) of bacterial composition at the genus level of the small intestine of mice described in (A). Data as mean±s.e.m. of n=10 (WT) or n=12 (*Acod1^-/-^*) mice from three experiments independently executed. ns: not significant (t-test).

**Supplementary Figure 8. Effects of itaconate treatment on fecal microbiota composition of HFD-fed mice.** (**A**) Levels of itaconate in stools of HFD-fed WT mice treated with 20 mM itaconate in drinking water for 12 weeks. Data as mean±s.e.m. of n=10 mice per group from two independent experiments independently executed. (**B**) Relative changes in the abundance of bacterial phyla in stools collected from WT mice treated as in (A) after 12 weeks of HFD-feeding. Data as mean±s.e.m. of n=10 mice per group from two experiments independently executed. (**C**) *Bacteroidetes* to *Firmicutes* ratio in the fecal microbiota of mice described in (B). Data as mean±s.e.m. of n=10 mice per group from two experiments independently executed. Fractional analysis (**D**) and relative changes (**E**) of the fecal bacterial composition at the family level of mice described in (B). Data as mean±s.e.m. of n=10 mice per group from two experiments independently executed. Fractional analysis (**F**) and relative changes (**G**) of the fecal bacterial composition at the genus level of mice described in (B). Data as mean±s.e.m. of n=10 mice per group from two experiments independently executed. (**H**) Relative changes in abundance of *Bacteroidetes* and *Firmicutes* phyla measured in fecal suspensions, deriving from pooled stools of n= 6 HFD-fed WT mice, incubated *in vitro* either with itaconate (0.5, 1, 10, 20 mM) or vehicle (-) for 48 hours in anaerobic atmosphere. Data as mean±s.e.m. from n=3 independent experiments. (**I**) *Bacteroidetes* to *Firmicutes* ratio measured in fecal suspensions described in (H). Data as mean±s.e.m. from n=3 independent experiments. Relative changes in the abundance of *Bacteroidaceae* (**J**) and *Bacteroides* (**K**) measured in fecal suspensions described in (H). Data as mean±s.e.m. from n=3 independent experiments. (**L**) Liver triglyceride content in *wild type* mice as treated in (A) after 16 weeks of HFD feeding. Data as mean±s.e.m. of n=5 (vehicle) and n=8 (itaconate)-treated mice from one representative experiment out of two independently executed. (**M**) qPCR analysis of the indicated genes in liver of *wild type* mice as described in (A) after 16 weeks of HFD feeding. Data as mean±s.e.m. of n=5 (vehicle) and n=8 (itaconate)-treated mice from one representative experiment out of two independently executed. (**N**) qPCR analysis of F4/80 (*Adgre1*) in fat depots of *wild type* mice as described in (A) after 16 weeks of HFD feeding. Data as mean±s.e.m. of n=5 (vehicle) and n=7 (itaconate)-treated mice from one representative experiment out of two independently executed. (**O**) Area under the curves (AUC) of ITT described in Fig. 5L. Data as mean±s.e.m of n=5 (vehicle) and n=7 (itaconate)-treated mice from one representative experiment out of two independently executed. (**P**) Food intake in HFD-fed WT mice as described in Fig.5J. Data as mean±s.e.m of n=5 (vehicle) and n=8 (itaconate)-treated mice from one representative experiment out of two independently executed. In (A-G), (L-P): **P*<0.05; ***P*<0.01; ****P*<0.001 (t-test). In (H-K): **P*<0.05; ***P*<0.01; ****P*<0.001 (one-way ANOVA followed by Dunnett`s T3 multiple comparison test compared with vehicle (-)); ns: not significant.

**Supplementary Figure 9. Fecal microbiota composition of mice used in FMT experiments before fecal transplantation.** Fractional analysis (**A**) and relative changes (**B**) of fecal bacterial composition at the phylum level of WT and *Acod1^-/-^* donor and recipient mice assessed before the fecal microbiota transplantation (FMT). Data as mean±s.e.m. of n=9 (WT donor), n=11 (*Acod1^-/-^* donor), n=8 (WT recipient), n=10 (*Acod1^-/-^* recipient) from two experiments independently executed. (**C**) Differential abundance (DESeq2) matrix comparing fecal bacterial composition at family level of taxa assigned to *Bacteroidetes* and *Firmicutes*. of mice described in (A). WT donor (n=9), *Acod1^-/-^* donor (n=11), WT recipient (n=8), *Acod1^-/-^* recipient (n=10) from two experiments independently executed. Fractional analysis (**D**) and relative changes (**E**) of fecal bacterial composition at the family level of mice described in (A). Data as mean±s.e.m. of n=9 (WT donor), n=11 (*Acod1^-/-^* donor), n=8 (WT recipient), n=10 (*Acod1^-/-^* recipient) from two experiments independently executed. (**F**) Differential abundance (DESeq2) matrix comparing fecal bacterial composition at genus level of taxa assigned to *Bacteroidetes* and *Firmicutes* in mice described in (A). WT donor (n=9), *Acod1^-/-^* donor (n=11), WT recipient (n=8), *Acod1^-/-^* recipient (n=10) from two experiments independently executed. Fractional analysis (**G**) and relative changes (**H**) of fecal bacterial composition at the genus level of mice described in (A). Data as mean±s.e.m. of n=9 (WT donor), n=11 (*Acod1^-/-^* donor), n=8 (WT recipient), n=10 (*Acod1^-/-^* recipient) from two experiments independently executed. (**I**) Abundance of bacteria in stools of WT (n=8) and *Acod1^-/-^* (n=11) recipient mice used in the FMT set-up measured before (-) and at the end (+) of antibiotics (ABX) treatment, determined by quantitative PCR-based measurement of bacterial 16S rRNA gene levels.

Data as mean±s.e.m. from two experiments independently executed. In (A), (B), (D), (E), (G), (H): one-way ANOVA followed by Dunnett`s T3 multiple comparison test compared with WT donor. In (I) **P*<0.05; ****P*<0.001 (t-test); ns: not significant.

**Supplementary Figure 10. Effect of FMT on fecal microbiota composition of HFD-fed mice.** Fractional analysis (**A**) and relative changes (**B**) of fecal bacterial composition at the phylum level of WT and *Acod1^-/-^* donor and recipient mice receiving either PBS (donors) or a fecal suspension from reciprocal genotypes (recipients) assessed after 18 weeks of feeding with HFD. Data as mean±s.e.m. of n=8 (WT donor), n=10 (*Acod1^-/-^* donor), n=8 (WT recipient), n=10 (*Acod1^-/-^* recipient) from two experiments independently executed. (**C**) Differential abundance (DESeq2) matrix comparing fecal bacterial composition at family level of taxa assigned to *Bacteroidetes* and *Firmicutes* of mice described in (A). WT donor (n=8), *Acod1^-/-^* donor (n=10), WT recipient (n=8), *Acod1^-/-^* recipient (n=10) from two experiments independently executed. Fractional analysis (**D**) and relative changes (**E**) of fecal bacterial composition at the family level of mice described in A. Data as mean±s.e.m. of n=8 (WT donor), n=10 (*Acod1^-/-^* donor), n=8 (WT recipient), n=10 (*Acod1^-/-^* recipient) from two experiments independently executed. (**F**) Differential abundance (DESeq2) matrix comparing fecal bacterial composition at genus level of taxa assigned to *Bacteroidetes* and *Firmicutes* in mice described in (A). WT donor (n=8), *Acod1^-/-^* donor (n=10), WT recipient (n=8), *Acod1^-/-^* recipient (n=10) from two experiments independently executed. Fractional analysis (**G**) and relative changes (**H**) of fecal bacterial composition at the genus level of mice described in A. Data as mean±s.e.m. of n=8 (WT donor), n=10 (*Acod1^-/-^* donor), n=8 (WT recipient), n=10 (*Acod1^-/-^* recipient) from two experiments independently executed. (**I**) Determination of itaconate levels in stools of mice described in (A). Data as mean±s.e.m. of n=9 (WT donor), n=11 (*Acod1^-/-^* donor), n=8 (WT recipient), n=10 (*Acod1^-/-^* recipient) from two experiments independently executed. In (A), (B), (D), (E), (G-I): **P*<0.05; ***P*<0.01; ****P*<0.001 (one-way ANOVA followed by Dunnett`s T3 multiple comparison test compared with WT donor). In (I) ^#^*P*<0.05 t-test; ns, not significant.

**Supplementary Figure 11. Fecal microbiota of *Acod1*-deficient mice offers transferable protection from metabolic disease by preventing meta-inflammation.**

(**A**) AUC of ITT described in Fig. 6C. Data as mean±s.e.m. of n=9 (WT donor), n=11 (*Acod1^-/-^* donor), n=8 (WT recipient), n=10 (*Acod1^-/-^* recipient) from two experiments independently executed. (**B**) mRNA levels of the indicated genes in colon of healthy (n=13) and obese (n=16) subjects (GSE158237). Boxes extend from 25th to 75th percentiles, black lines=median, whiskers=minimum to maximum. (**C**) qPCR analysis of the indicated genes in BAT of mice as described in Fig. 6A; n=7 (WT control), n=9 (*Acod1^-/-^* control), n=7 (WT and *Acod1^-/-^* recipient) mice from two experiments independently executed. In (A) and (C) one-way ANOVA followed by Dunnett's multiple comparisons test, compared with WT control mice. In (B) t-test. **P*<0.05; ***P*<0.01; ****P*<0.001; ^#^*P*<0.05; ns, not significant.

**Supplemental Methods and Materials.**

**RNA-sequencing (RNA-seq).** After RNA extraction, RNA integrity was confirmed by capillary electrophoresis (Fragment Analyzer, Agilent). Barcoded mRNA Libraries were prepared using NEBNext RNA Ultra II Directional Library Kit (New England Biolabs), following the manufacturer’s instructions. Sequencing was performed on an Illumina NovaSeq instrument. The 3' adaptors were removed using cutadapt and trimmed reads with a length of less than 18bp were discarded. The trimmed reads were filtered with a contaminants database. The recovered non matching reads were aligned to the genome/transcriptome using STAR-align (v 2.4.2a) to the GENCODE reference genome build GRCm38.95. Genes with very low read counts were excluded from downstream analysis. The minimum threshold was set at 1 count per million mapped reads across all samples per tissue. Gene count tables were loaded into R (v 4.1.0) and differential gene expression analysis was performed using the DESeq2 workflow[67]. Row normalized z-scores of transcripts per million (TPMs) were used to plot the heatmaps.

**Liver Proteomics.** Frozen liver samples were powderized in liquid nitrogen using a ceramic mortar and pestle and lyophilized. Weighed tissue (approximately 2mg of each) was homogenized in freshly prepared high-salt buffer (50mM Tris-HCl, 3M NaCl, 25mM EDTA, 0.25% w/v CHAPS, pH 7.5) containing 1x protease inhibitor (Halt Protease Inhibitor, Thermo Scientific) at a concentration of 10mg/mL. Homogenization took place in a bead beater (Bullet Blender Storm 24, Next Advance, 1mm glass beads) for 3min at 4^º^C. Samples were then spun for 20min at 18,000g at 4^º^C and the supernatant removed and stored as *Fraction 1*. A fresh aliquot of high-salt buffer was added to the remaining pellet at 10mg/mL of the starting weight, vortexed at 4^º^C for 15min, and spun for 15min. The supernatant was removed and stored as *Fraction 2.* This high-salt extraction was repeated once more to generate *Fraction 3,* after which freshly prepared guanidine extraction buffer (6 M guanidinium chloride adjusted to pH 9.0 with NaOH) was added at 10mg/mL and vortexed for 1h at RT. The samples were then spun for 15min, the supernatant removed, and stored as *Fraction 4. Fractions 1, 2, & 3* were combined, and all fractions were stored at -20^º^C until further analysis. Samples were digested according to the FASP protocol using a 10kDa molecular weight cutoff filter. In brief, 50ul of the samples were mixed in the filter unit with 8 Murea, 100mM ammonium bicarbonate (AB) pH 8.0, and centrifuged at 14 000*g* for 15min. The proteins were reduced with 10mM DTT for 30min at RT, centrifuged, and alkylated with 55mM iodoacetamide for 30min at RT in the dark. Following centrifugation, samples were washed 3× with urea solution, and 3× with 50 mM AB, pH 8.0. Protein digestion was carried out with sequencing grade modified Trypsin (Promega) at 1/50 protease/protein (wt/wt) at 37°C overnight. Peptides were recovered from the filter using 50mM AB. Samples were dried in Speed-Vac and desalted and concentrated on Thermo Scientific Pierce C18 Tip. A 20ul of each sample was loaded onto individual Evotips for desalting and then washed with 20μL 0.1% FA followed by the addition of 100μL storage solvent (0.1% FA) to keep the Evotips wet until analysis. The Evosep One system (Evosep, Odense, Denmark) was used to separate peptides on a Pepsep column, (150μm inter diameter, 15cm) packed with ReproSil C18 1.9 um, 120A resin. The system was coupled to the timsTOF Pro mass spectrometer (Bruker Daltonics, Bremen, Germany) via the nano-electrospray ion source (Captive Spray, Bruker Daltonics). The mass spectrometer was operated in PASEF mode. The ramp time was set to 100 ms and 10 PASEF MS/MS scans per topN acquisition cycle were acquired. MS and MS/MS spectra were recorded from *m/z* 100 to 1700. The ion mobility was scanned from 0.7 to 1.50 Vs/cm^2^. Precursors for data-dependent acquisition were isolated within ± 1 Th and fragmented with an ion mobility-dependent collision energy, which was linearly increased from 20 to 59 eV in positive mode. Low-abundance precursor ions with an intensity above a threshold of 500 counts but below a target value of 20000 counts were repeatedly scheduled and otherwise dynamically excluded for 0.4 min. MS/MS spectra were extracted from raw data files and converted into .mgf files using MS Convert (ProteoWizard, Ver. 3.0). Peptide spectral matching was performed with Mascot (Ver. 2.5) against the Uniprot mouse database (release 201701). Mass tolerances were +/- 15ppm for parent ions, and +/- 0.4 Da for fragment ions. Trypsin specificity was used, allowing for 1 missed cleavage. Met oxidation, Pro hydroxylation, protein N-terminal acetylation, and peptide N-terminal pyroglutamic acid formation were set as variable modifications with Cys carbamidomethylation set as a fixed modification. Scaffold (version 4.6, Proteome Software, Portland, OR, USA) was used to validate MS/MS based peptide and protein identifications. Peptide identifications were accepted if they could be established at greater than 95.0% probability as specified by the Peptide Prophet algorithm. Protein identifications were accepted if they could be established at greater than 99.0% probability and contained at least two identified unique peptides. Total peptide hits were normalized via auto scale normalization and statistical analysis was performed using MetaboAnalyst 5[68] in order to assess differentially expressed proteins between biological groups. Row normalized z-scores of peptide hits were used to plot the heatmaps.

**Functional Enrichment Analyses.** Over-representation analyses (ORA) of RNA-sequencing and proteomics data were performed with Shiny-GO 0.76[69] by using Gene Ontology (GO) terms[70] and KEGG[71]. Only differentially expressed genes/proteins in response to HFD and rescued by *Acod1* loss with an adjusted *P* value (FDR) <0.1 in both comparisons were considered. No threshold was set on the fold change as some of the genes/proteins might show moderate, yet biologically relevant changes.

**Metabolite extraction and LC-MS.** For metabolite determination, 40 mg of frozen tissue was dissociated in 1ml of ice-cold extraction solution, composed of methanol, acetonitrile, and water (5:3:2), using ceramic beads and a Precellys tissue homogenizer (Bertin Instruments) by 3 cycles of oscillations at 8000 RPM (45 seconds each paused by 30-second intervals) at 4^º^C. After centrifugation (16,000g for 30min at 4°C), the supernatants were analyzed by liquid chromatography-mass spectrometry (LC-MS) by using a Q Exactive Orbitrap mass spectrometer (Thermo Fisher Scientific) coupled with a Thermo Fisher Scientific Accela HPLC system, as previously described[72]. Data were acquired and analyzed with Thermo Xcalibur and Thermo LCquan 2.7 (Thermo Fisher Scientific) software, respectively. Absolute quantification of itaconate levels in stools of mice was determined by sample peak areas comparison to reference standards.

For measurements of free fatty acids in liver samples, an ultra-high pressure-liquid chromatography-mass spectrometry (UHPLC-MS – Vanquish and Q Exactive, Thermo Fisher) was used as previously reported[73]. Briefly, liver samples were extracted in ice cold methanol:acetonitrile:water (5:3:2 v/v/v) at a concentration of 15mg/mL. Glass beads (1.0mm, BioSpec Products Inc. cat.# 11079110) were added to each sample and samples were placed in the bead beater (GenoGrinder; SPEX, Metuchen, NJ) for 5 minutes at level 5 at 4°C. Samples were then vortexed for 30min at 4°C and centrifuged at 18,213 g for 10min at 4°C. Supernatants were processed for metabolomics analyses. Five microliters of sample extracts (diluted to 15mg/ml in extraction solution) were loaded onto a Kinetex XB-C18 column (150 × 2.1 mm i.d., 1.7 μm – Phenomenex). A 5 min gradient (5%–95% B, phase A: water + 0.1% formic acid and phase B: acetonitrile with + 0.1% formic acid for positive ion mode; 0%–100% B, phase A: 5% acetonitrile + 5mM ammonium acetate and phase B: 95% acetonitrile + 5mM ammonium acetate for negative ion mode) were used to elute metabolites. The mass spectrometer scanned in Full MS mode at 70,000 resolution in the 65–975 m/z range, 4 kV spray voltage, 45 sheath gas and 15 auxiliary gas, operated in negative and then positive ion mode (separate runs). Fatty acids assignment was performed against an in-house standard library, as reported[73, 74].

**Diglyceride profiling.** Liver samples were collected and diglyceride profiling was performed via ultra-high pressure-liquid chromatography-mass spectrometry (UHPLC-MS – Vanquish and Q Exactive, Thermo Fisher Scientific). Briefly, liver samples were extracted in 100% ice cold methanol at a concentration of 15mg/mL of buffer. Glass beads were added to each sample and samples were placed in the bead beater (Bullet Blender Storm 24, Next Advance, 1mm glass beads) for 5 minutes at level 5 at 4°C. Samples were then vortexed for 30min at 4°C and then centrifuged at 18,213 g for 10 min at 4°C. 40uL of the sample extract and 40uL of 10mM ammonium acetate were then added to an autosampler vial. Five microliters of sample extracts were loaded onto a Acquity HSS T3 column (150 x 2.1mm i.d., 1.8μm). A 17 min gradient (25%-99% B, phase A: 75:25 water:acetonitrile + 5mM ammonium acetate, phase B: 50:45:5 isopropanol:acetonitrile:water + 5mM ammonium acetate) was used to elute the metabolites. The mass spectrometer scanned in Full MS mode at 70,000 resolution in the 150-1500 m/z range, 4 kV spray voltage, 45 sheath gas and 15 auxiliary gas, operated in negative mode. Metabolite assignment was performed against an in-house standard library, as reported[73, 74].

**Measurement of liver triglycerides and NH_3_**. Triglycerides and NH_3_ levels were determined colorimetrically in 10mg of frozen liver tissues by using the Triglyceride Assay Kit (ab65336, Abcam) and Ammonia Assay Kit (ab83360, Abcam), respectively, following manufacturer's instructions.

**SCFA measurement.** For short chain fatty acids (SCFA) measurement, 10mg of stools were homogenized in 400µl methanol-water (1:1, v/v) using ceramic beads and a Precellys tissue homogenizer (Bertin Instruments) by 3 cycles of oscillations at 8000 RPM (45 seconds each paused by 30-second intervals) at 4^º^C. After centrifugation (16,000g for 15min at 4 °C), 200µl of the supernatants were transferred to a new microfuge tube and dried by SpeedVac. Dried tissue extracts were resuspended in 240µl of water and derivatized as previously reported[75]. Briefly, each sample was mixed with 50µl of 1-propanol (34871, Sigma-Aldrich) and 50µl of pyridine (360570, Sigma-Aldrich) and incubated on ice for 5min. Then, 100 µl of 1 M sodium hydroxide was added, immediately followed by 30µl of methyl chloroformate (M35304, Sigma-Aldrich) and vigorous vortexing for 20s. Then, each sample was mixed with 300µl of methyl tert-butyl ether (650560, Sigma-Aldrich), vortexed for another 20 sec and centrifuged at 10,000g for 5min. 200μL microliters of the resulting upper layer was transferred to a gas-chromatography (GC) vial for analysis. Propyl derivatives of propionate and butyrate were analysed using an Agilent 7000 GC/MS provided with an Agilent 7890 GC chromatograph and 7693 autosampler (Agilent, CA, United states). High purity helium (99.999%) was the carrier gas with a column head pressure of 16.3 psi. The total flow and the septum purge flow rates were 24.9ml/min and 3mL/min, respectively. Injector was set at a temperature of 280 °C. Injection was made at an injection volume of 1µL in the split mode with a split ratio of 10:1. The chromatographic separation was performed on a Zebron ZB-1701P GC capillary column 30m X 0.25mm X 0.25μm film thickness (Cat. No. [7HG-G012-11](https://dev.phenpreview2.com/en/Products/Zebron-gc-columns/Zebron-ZB-1701P##), Phenomenex, CA, U.S.A). The oven temperature was set to 70°C initial temperature held for 2 min followed by a linear increase to 140°C at 10°C/min, followed by a more rapid increase to 182°C (held for 1 min) at 20°C/min then to 280°C at 50°C/min. The total run time was 16 minutes. The MS was operated in [electron ionization](https://www.sciencedirect.com/topics/biochemistry-genetics-and-molecular-biology/electron-ionization) (EI) mode at 70 eV and the interface was set to 300°C.  Data was acquired in full scan made over the range of m/z = 25-200 with a scan time of 300ms. MS recording started at a cut-off of 1min. Data processing was performed using Agilent MassHunter Quantitative analysis software (Agilent, CA, United states). Initial compound annotation was performed by searching against NIST11 mass spectral library (National Institute of Standards and Technology, Gaithersburg, MD). The identity of the propyl derivatives was further confirmed by comparison to reference standards using a combination of retention time and fragmentation pattern. Selected fragments were used for the quantitation of the propyl derivatives, which were 75.1 and 71.1 for propyl propionate and butyrate, respectively. The respective retention times were 3.55, and 4.95min.

**Histology and image analyses.** Formalin-fixed (HT501128, Sigma-Aldrich) paraffin-embedded sections were dewaxed and hydrated through graded decrease alcohol series. For histological analysis, slides were stained using standard protocols for Hematoxylin and Eosin (Mayer’s Hematoxylin, BioOptica #05-06002/L and Eosin, BioOptica #05-10002/L). For immunohistochemistry (IHC), slides were immunostained with Automatic Leica BOND RX system (Leica Microsystems GmbH, Wetzlar, Germany). Antigen-retrieval was performed using sodium citrate buffer at 100°C. Primary antibodies anti-F4/80 (dilution 1/500; F4/80 (D2S9R), Cell Signaling Technology, Inc.) and anti-UCP1 (1/500; ab10983, Abcam) were developed with Bond Polymer Refine Detection (Leica, DS9800). Bright-field images were acquired with an Aperio AT2 digital scanner (Leica Biosystems) and Aperio ImageScope software (v12.4.3.5008, Leica Biosystem). Image analysis was performed using ImageJ. Lipid droplet particles were determined using thresholding and by applying limits for particle size and circularity to avoid inclusion of non-lipid droplet structures (n=3-4 per group with an average of 10 images per tissue). Adipocyte size from WAT was determined using the Adiposoft[76] plugin (n=5 per group with an average of 5 images per tissue). After color deconvolution F4/80 positive areas were quantified by binarization (n=3 per group with an average of 5 images per tissue). In BAT, UCP1 positive areas were determined by thresholding and normalized to the cell number (n=3 per group with an average of 3 images per tissue) per field of view.

**Glycemia and insulinemia determination.** Blood was collected from the tail of overnight-fasted mice. Then, glucose and insulin levels were determined by using a handheld glucometer (Contour XT Meter, Ascensia Diabetes Care Italy) and a precoated ELISA Kit (EMINS, Invitrogen), respectively, according to the manufacturer’s instructions.

**Glucose and insulin tolerance test.** For glucose-tolerance test (GTT), mice were fasted overnight before receiving an intraperitoneal injection of glucose solution (2g of glucose per kg body weight). For insulin tolerance test (ITT), mice received an intraperitoneal injection of insulin solution (0.75U of insulin per kg body weight) after 6-hours fasting. For both GTT and ITT, glycemia was determined at the indicated time points as above described.

**Indirect calorimetry and activity measurements.** Measurements of oxygen consumption (VO_2_), carbon dioxide (CO_2_) production, respiratory exchange ratio, energy expenditure, food intake and locomotor activity were performed using open-circuit indirect calorimetric chambers (PhenoMaster, TSE Systems, Bad Homburg, Germany). Mice were individually caged, fed with a HFD and acclimated to the chambers for 36h before experimental recordings. Calorimetric data show the average of 6 days or 6 nights. Energy expenditure and VO_2_ were calculated using the manufacturer’s software and values were corrected for body mass. Total locomotor activity was measured using a 3-dimensional infrared light-beam.

**Determination of total fecal energy excretion.** Fecal energy content, used as a proxy for intestinal nutrient absorption capacity, was determined in 0.5g of frozen stools isolated from HFD-fed mice by bomb calorimetry (Phenomin facility).

**16s ribosomal DNA gene sequencing and analysis.** Total DNA was extracted from fecal samples of ND and HFD-fed mice using the QIAamp PowerFecal Pro DNA Kit (Qiagen), following manufacturer's instructions. The V3-V4 region of the 16s rRNA gene was amplified starting from 500ng of extracted DNA using the AccuPrime Taq DNA Polymerase (Invitrogen), the following primers: V3-16S-Fw: TCG TCG GCA GCG TCA GAT GTG TAT AAG AGA CAG CCT ACG GGN GGC WGC AG; V4-16S-Rev: GTC TCG TGG GCT CGG AGA TGT GTA TAA GAG AGA CAG GAC TAC HVG GGT ATC TAA TCC; and the amplification protocol: 94°C for 2 min., 35 cycles of 94°C for 30sec, 56°C for 30sec, and 68°C for 1min and finally stored at 4°C. Amplicons were purified using the AMPure XP beads (Beckman Coulter, Brea, USA). A second PCR step was performed for indexing and add Illumina sequencing adapters to each sample. The Nextera XT Index Kit (Illumina) and the KAPA HiFi HotStart PCR Kit (KAPA Biosystem) were used following the amplification protocol: 95°C for 3min, 8 cycles of 95°C for 30sec, 55°C for 30sec, 72°C for 30sec, 72°C for 4min and then stored at 4°C. A second purification step with AMPure XP beads was performed to clean up the samples for preparing the final library. Purified DNA is quantified using the Qubit double-stranded DNA (dsDNA) HS Assay Kit on a Qubit 2.0 Fluorometer (Thermo Fisher) and then diluted and pooled following Illumina protocol. Sequencing is performed using the MiSeq Illumina platform with a 600 (2 X 300)-base pairs (bp) paired-end read protocol.

For the metagenomic profiling of the small intestines isolated from HFD-fed *wild type* and *Acod1^-/-^* mice, total DNA was extracted from small intestine samples of mice using the DNeasy Blood & Tissue Kits (Qiagen), following the manufacturer's instructions with these modifications: 180 uL of ATL and 20 uL of Proteinase K were added to a small piece of intestine and incubated for 10h at 56°C at 600 RPM in a thermomixer compact (Eppendorf). The DNA was lastly eluted in 60 uL of Buffer AE after an incubation of 5 minutes. Then, the V3-V4 region of the 16s rRNA gene was amplified starting from 500 ng of extracted DNA as described above. Amplicons were purified and sequenced using the MiSeq Illumina platform with a 600 (2 X 300)-base pairs (bp) paired-end read protocol as described above.

For all metagenomics analyses, FastQ files were processed using the Divisive Amplicon Denoising Algorithm 2 (DADA2)[77] pipeline (version 1.26.0) to de-noise the sequences, remove chimeras and assign amplicon sequence variants (ASVs) using standard quality filter parameters (maxN=0, maxEE=2, truncQ=2, rm.phix=TRUE) and trimming 25 and 75 bases of the 3′ region of the forward and reverse reads, respectively. Taxonomic classification was performed implementing a naive Bayesian classifier approach using the Greengenes 13.8 database. Lowly abundant microbial taxa with a relative abundance of less than 0.01% in less than 75% of the samples were excluded from downstream analyses. Differential abundance analysis was performed using the DESeq2 workflow[67] on taxonomic levels and accurate estimation of log-fold changes was performed using the apeglm shrinkage estimator (version 1.20.0)[78].

**Treatment of fecal bacterial cultures with itaconate.** Bacterial cultures were performed starting from fecal pool derived from 6 HFD-fed *wild type* mice. Each pool was resuspended and homogenized in 1.5 mL of BBL™ Brain Heart Infusion (BHI) culture medium (Becton, Dickinson and Company, BD) and then centrifuged for 2 minutes at 840Xg with Prism™ microcentrifuge (Labnet). One hundred microliters of obtained supernatants were transferred in four BHI vials containing 0.5, 1, 10 and 20 mM itaconate, respectively. One BHI vial supplemented with phosphate saline buffer was used as control of bacterial growth. All vials were placed at 37 °C in AnaeroJar 2.5 L Jar System (OXOID) and using Thermo Scientific™ Oxoid™ AnaeroGen™ 2.5L Sachet (Fisher Scientific) in order to generate an anaerobic atmosphere. Three samplings of 500 µL were carried out every two days from each vials and, finally, extraction of genetic material and sequencing were performed as described above.

**Treatment of pure bacterial cultures with itaconate.** The antimicrobial effect of the itaconate was tested on five pure bacteria strains belonging to *Bacteroidaceae* family (*Bacteroides* genus) obtained from DSMZ: *Bacteroides acidifaciens* (DSM 15896), *Phocaeicola vulgatus* (DSM 1447), *Phocaeicola dorei* (DSM 17855), *Bacteroides thetaiotaomicron* (DSM 2079) and *Bacteroides uniformis* (DSM 6597). Briefly, the freeze-dried bacteria pellets were rehydrated using 500ul of BBL™ Brain Heart Infusion (BHI) culture medium (Becton, Dickinson and Company, BD). To identify a single culture medium suitable for all the strains of interest, preliminary experimental growths were performed using 3 liquid media (BHI, Columbia, and Columbia supplemented with 5% defibrinated sheep blood). The Columbia medium allowed the growth of all bacteria tested and therefore it was chosen for next experiments. To test the effects of itaconate on the different strains, all bacteria were cultured in Columbia medium and 24h after colony inoculum, 50uL of bacterial suspensions were grown in 5mL of fresh Columbia medium either in the presence or absence of 0.5mM itaconate for further 24h at 37°C in anaerobic condition (in AnaeroJar 2.5 L Jar System (OXOID) with Thermo Scientific™ Oxoid™ AnaeroGen™ 2.5L Sachet (Fisher Scientific)). At the end of the experiment, bacteria abundance in all cultures was determined spectrophotometrically by measuring absorbance (OD) at 600 nm by the Multiskan Go, Thermo Scientific™ (Fisher Scientific).

**Plasma metabolic hormones and cytokines determination.** Circulating levels of GLP-1, resistin, Leptin, TNF, IL-1B, and IL6 were determined by multiplex bead-based assay (Eve Technologies, Calgary, AB, Canada).

**Measurement of intestinal permeability.** Gut permeability was determined using the fluorescein isothiocyanate (FITC)–dextran assay. In brief, mice fed with HFD for 18 weeks were fasted for 4h and orally gavaged with 300mg/kg 4kDa FITC dextran (FD4-100MG Sigma-Aldrich) solubilized in sterile PBS. Four hours later, approximately 80µl of blood was collected and the relative levels of FITC dextran in plasma diluted 1:10 in PBS were measured by fluorescence spectroscopy (excitation at 485nm and emission at 535nm).

**Measurement of fecal bacterial abundance.** The content of bacteria in stools of mice was determined by quantitative PCR of the gene encoding bacterial 16S rRNA[79]. Briefly, DNA was extracted from equal amount of fecal material derived from *wild type* and *Acod1^-/-^* mice before and at the end of antibiotics treatment, by the QIAamp PowerFecal Pro DNA Kit (Qiagen) following the manufacturer’s instructions. For real-time qPCR analysis, the variable regions 3 and 4 (V3–V4) of 16S ribosomal ribonucleic acid (rRNA) gene were amplified starting from extracted genetic material. Amplification primers used were 16S_FW: TCG TCG GCA GCG TCA GAT GTG TAT AAG AGA CAG CCT ACG GGN GGC WGC AG and 16S_Rev: GTC TCG TGG GCT CGG AGA TGT GTA TAA GAG ACA GGA CTA CHV GGG TAT CTA ATCC. SYBR Green PCR Master mix (Applied Biosystem) and the following cycling conditions were used: 95°C for 2 min, 45 cycles of (95C for 20’’, 56°C for 20’’ and 72°C for 30’’), 72° for 10 min. qPCRs were carried out using a 7900HT Fast Real-Time PCR System (Applied Biosystem). Serial dilutions of DNA from the E. coli DH5a strain were used as standard for absolute quantification of bacterial load in each sample.

**Fecal microbiota transplantation.** For fecal microbiota transfer (FMT), 5-week old male *wild type* and *Acod1^-/-^* littermates were randomly divided in two groups: donors and recipients. Before the transplantation, recipient mice only were treated with a broad-spectrum antibiotic cocktail (50 μg/ml Streptomycin, 100 U/ml Penicillin) in drinking water for 3 days to lower existing microbiota and favour the following engraftment of donor microbiota, as previously described[80]. Soon after antibiotics removal, HFD feeding was started for all mice. Starting from the first day of HFD administration and throughout the whole experiment (18 weeks), recipient *wild type* and *Acod1^-/-^* mice were gavaged daily (4 consecutive times per week) with 200µl of a fecal slurry prepared by pooling stools freshly-collected from their counterparts of reciprocal genotype in the donor group. As control, donor mice instead received 200µl of sterile PBS by oral gavage daily (4 consecutive times per week). In detail, fecal slurries were prepared by grinding stools pooled from either *wild type* or *Acod1^-/-^* donor mice in sterile PBS at the concentration of 100mg/ml. The slurries were then centrifuged at 300g for 2 min to discard insoluble materials and the supernatants used for gavaging recipient mice, as above described. Collection of stools, preparation of fecal slurries and mouse gavaging procedures were conducted under sterile conditions (under laminar flow hood).

***In vitro* adipogenesis assay.** Pre-adipocytes were isolated from iWAT collected from 8-week old *wild type* and *Acod1^-/-^* mice fed with normal diet and digested in DMEM/F12 (D8437, Merck) supplemented with 100U/ml penicillin, 100mg/ml streptomycin and 1mg/ml collagenase IV (17104-019, Gibco) for 30min at 37°C by shaking. The cell suspension was filtered through a 70μm cell strainer and centrifuged 8min at 500g. Supernatant was removed and the pellet incubated with ACK Lysing Buffer (A10492-01, Gibco) for 5min at RT. Pre-adipocytes were resuspended in DMEM/F12 supplemented with 10% FBS, 4mM L-glutamine, 100U/ml penicillin and 100mg/ml streptomycin, seeded in 24-well plates (30,000 cells per well) and grown to confluence. Culture medium was replaced with differentiation medium containing 1µM dexamethasone (190040, MP Biomedicals), 0.5mM IBMX (I5879, Merck), 1µM rosiglitazione (R2408, Merck) and 5µg/ml insulin (Merck, I9278). After 72h, medium was replaced with medium containing 1µM rosiglitazione and 5µg/ml insulin. After 5 days cells were washed with PBS and lysed for RNA extraction or fixed with formalin solution (HT501128, Sigma-Aldrich) for 15min and stained with 0.3% Oil-Red-O solution (O1391, Sigma-Aldrich) for 15min. Air dried samples were destained with isopropanol and absorbance was measured at 490nm using a microplate reader (Bio-Rad) to determine lipid content.

**Generation and treatment of BMDMs.** Bone marrow (BM)-derived macrophages (BMDMs) were prepared from 5 week-old CO_2_-euthanized C57BL/6N *wild type* and *Acod1*^-/-^ mice as previously reported[72]. At the end of the differentiation, cultured BMDMs were stimulated with 100 ng/ml LPS (Enzo Life Sciences) for 24h or with 2.5mM ATP for 40min after priming with LPS for 24h for inflammasome activation.

**Enzyme-linked immunosorbent assay (ELISA).** IL-1β concentrations and IL-6 levels in BMDMs culture medium were determined using IL-1 beta (88701376, Invitrogen) or IL-6 (88-7064-22, Invitrogen) mouse-uncoated ELISA Kits, respectively, according to the manufacturer’s instructions. Absorbance was measured at a wavelength of 450nm, subtracting the values measured at 570nm, using a microplate reader (Bio-Rad).
