# Supplementary figures and images for: ACOD1 deficiency offers protection in a mouse model of diet-induced obesity by maintaining a healthy gut microbiota
Source: Cell Death Dis. 2024 Feb 1;15(2):105. doi: 10.1038/s41419-024-06483-2 (PMC10834593; doi:10.1038/s41419-024-06483-2)

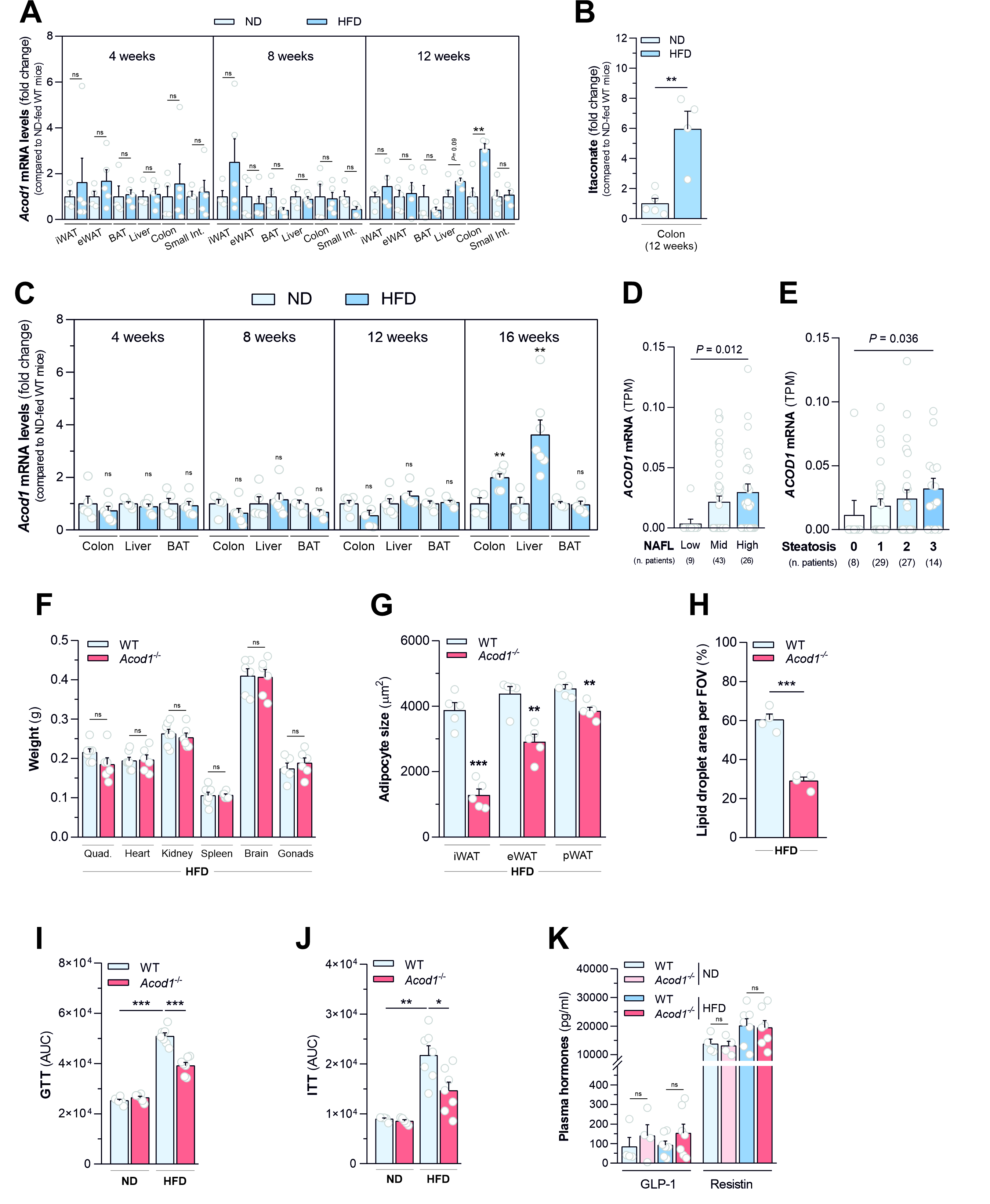

Supplement: Supplementary file 2 — Supplementary Figure 1 [file 41419_2024_6483_MOESM2_ESM.jpg]

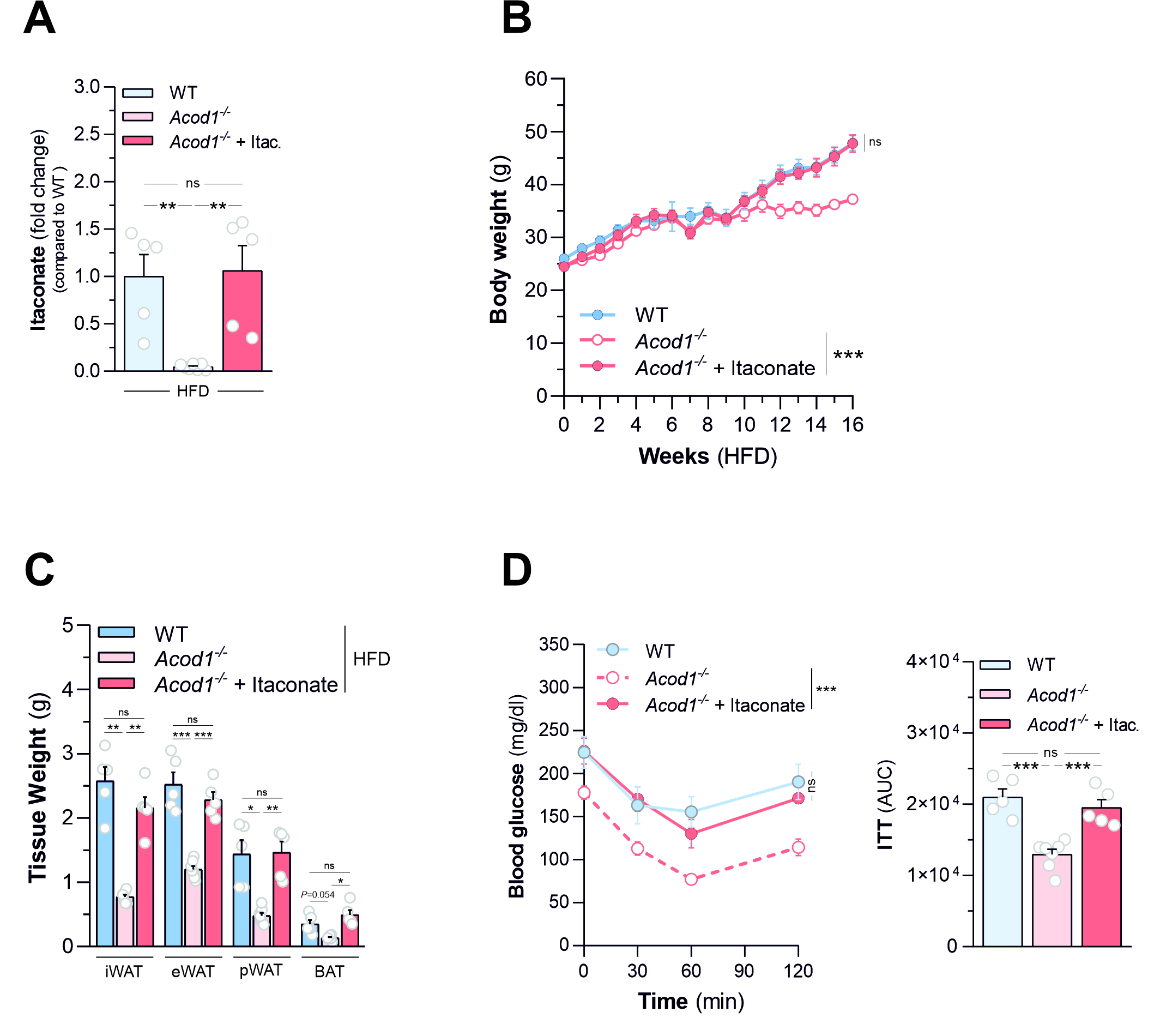

Supplement: Supplementary file 3 — Supplementary Figure 2 [file 41419_2024_6483_MOESM3_ESM.jpg]

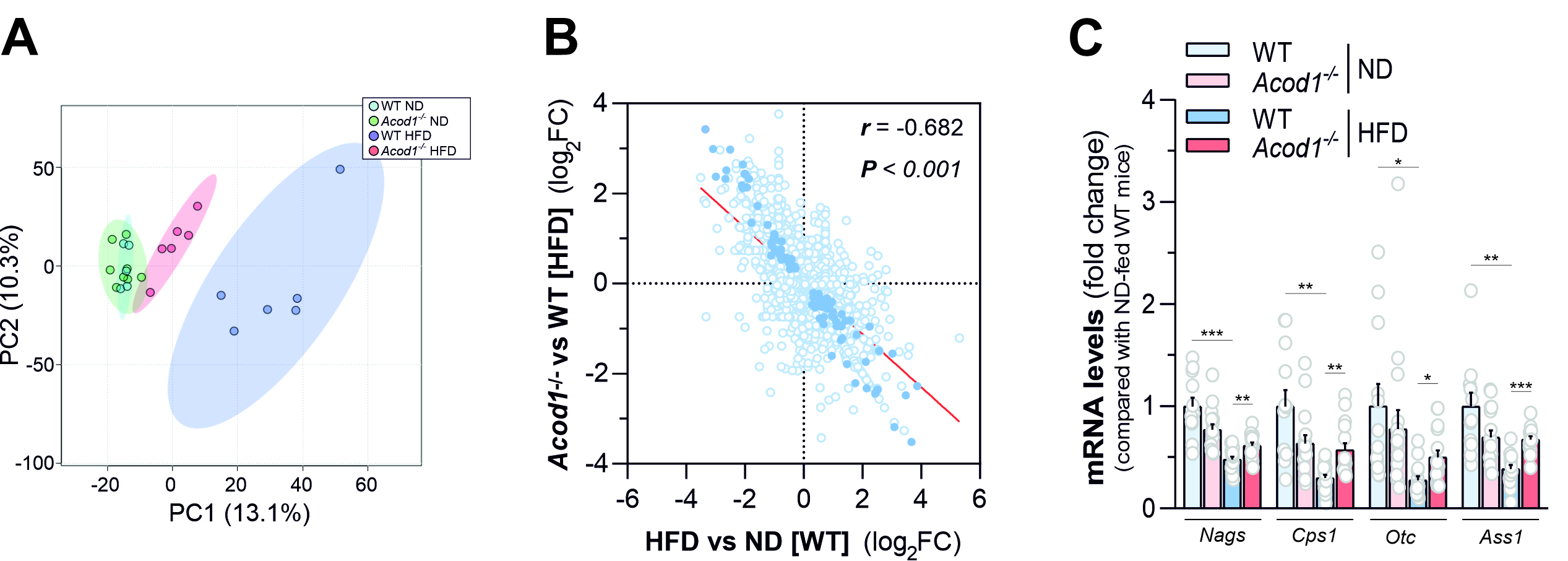

Supplement: Supplementary file 4 — Supplementary Figure 3 [file 41419_2024_6483_MOESM4_ESM.jpg]

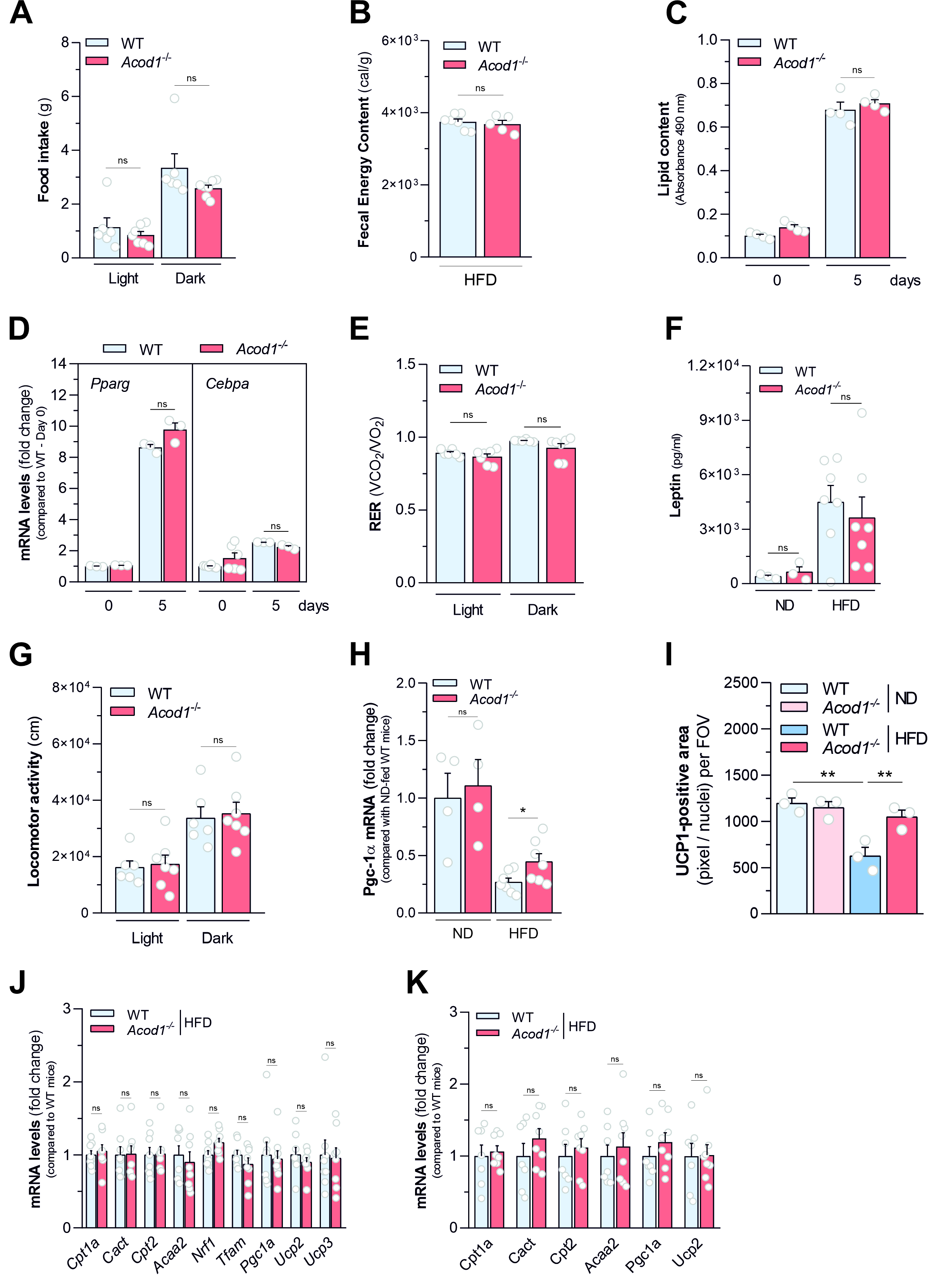

Supplement: Supplementary file 5 — Supplementary Figure 4 [file 41419_2024_6483_MOESM5_ESM.jpg]

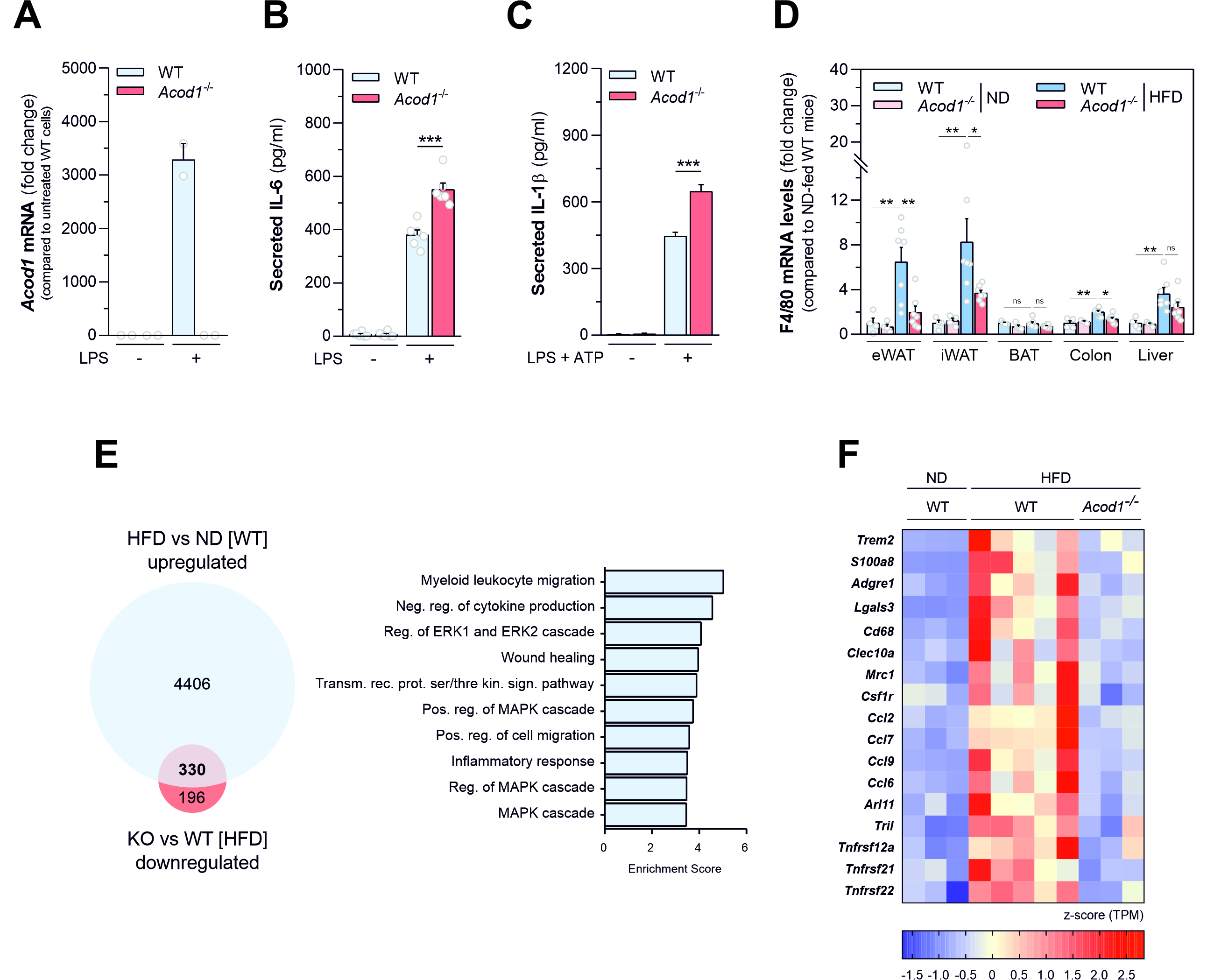

Supplement: Supplementary file 6 — Supplementary Figure 5 [file 41419_2024_6483_MOESM6_ESM.jpg]

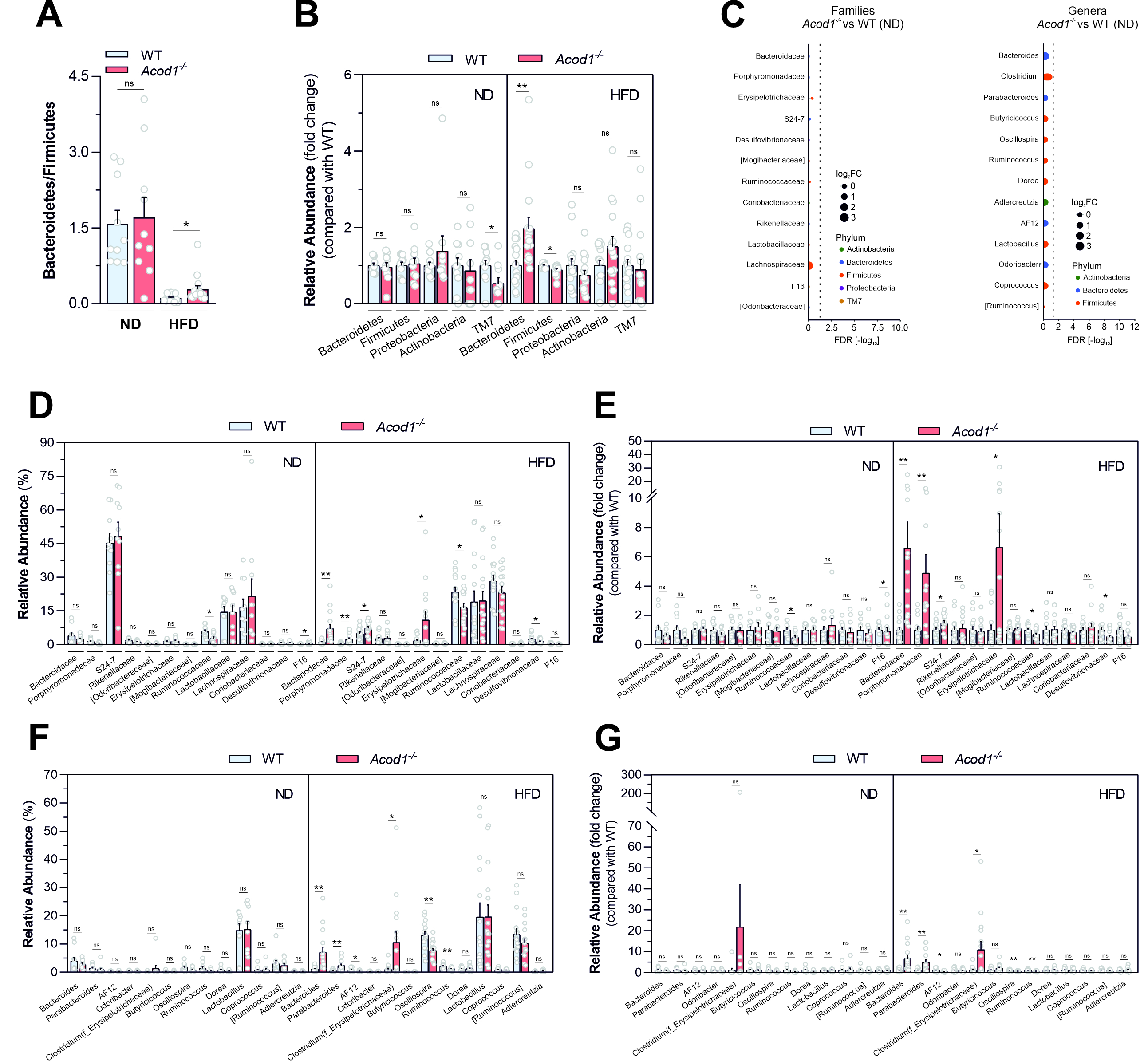

Supplement: Supplementary file 7 — Supplementary Figure 6 [file 41419_2024_6483_MOESM7_ESM.jpg]

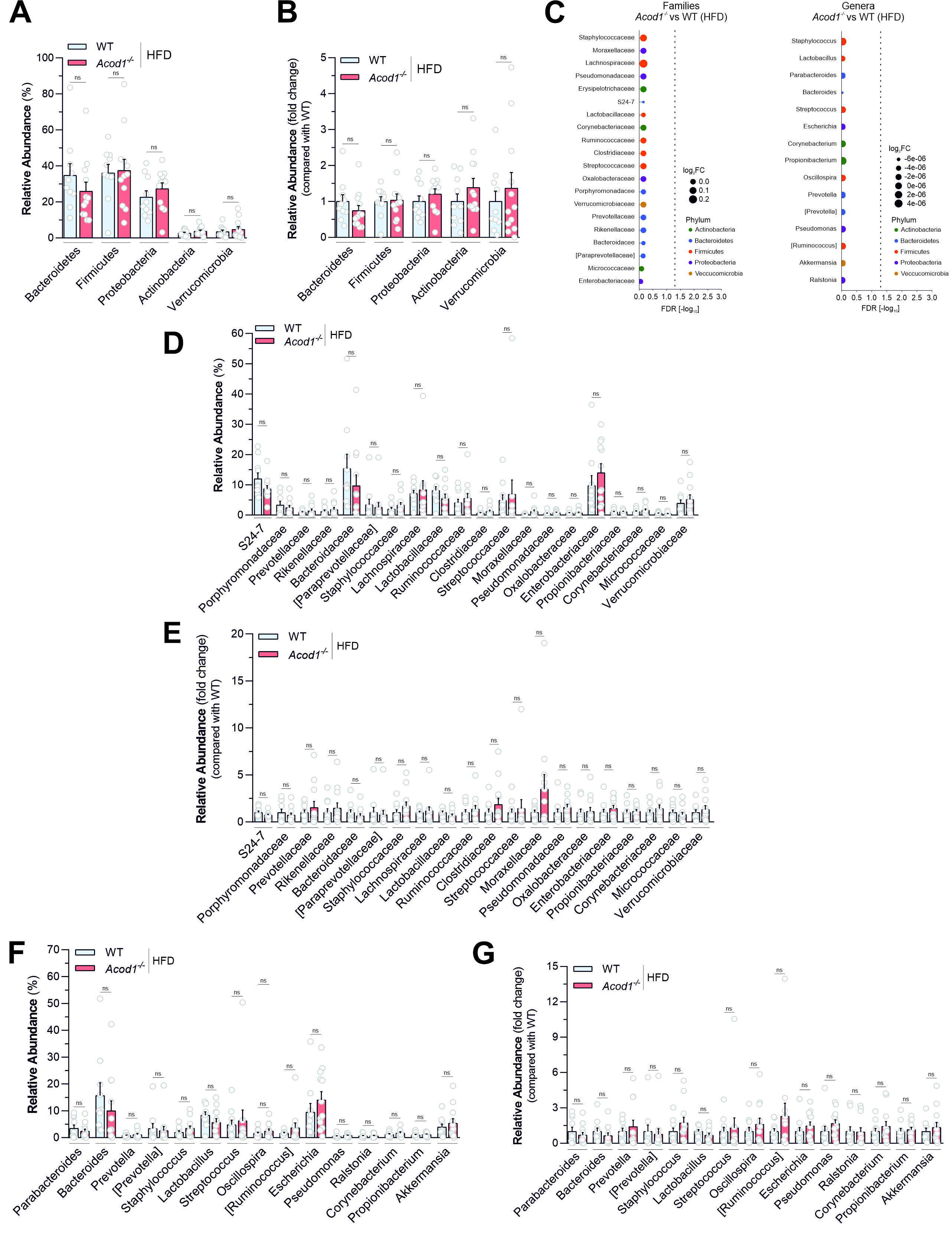

Supplement: Supplementary file 8 — Supplementary Figure 7 [file 41419_2024_6483_MOESM8_ESM.jpg]

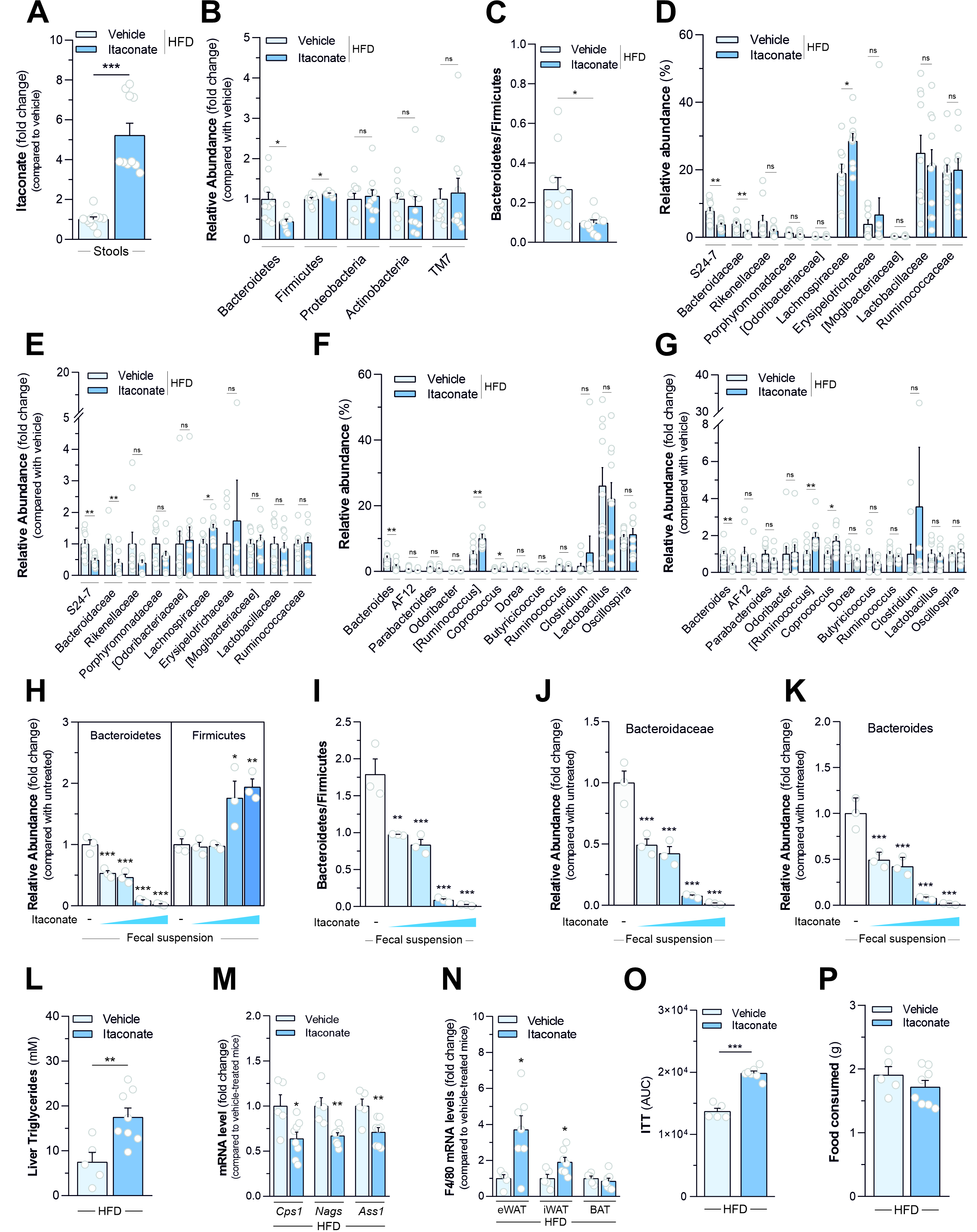

Supplement: Supplementary file 9 — Supplementary Figure 8 [file 41419_2024_6483_MOESM9_ESM.jpg]

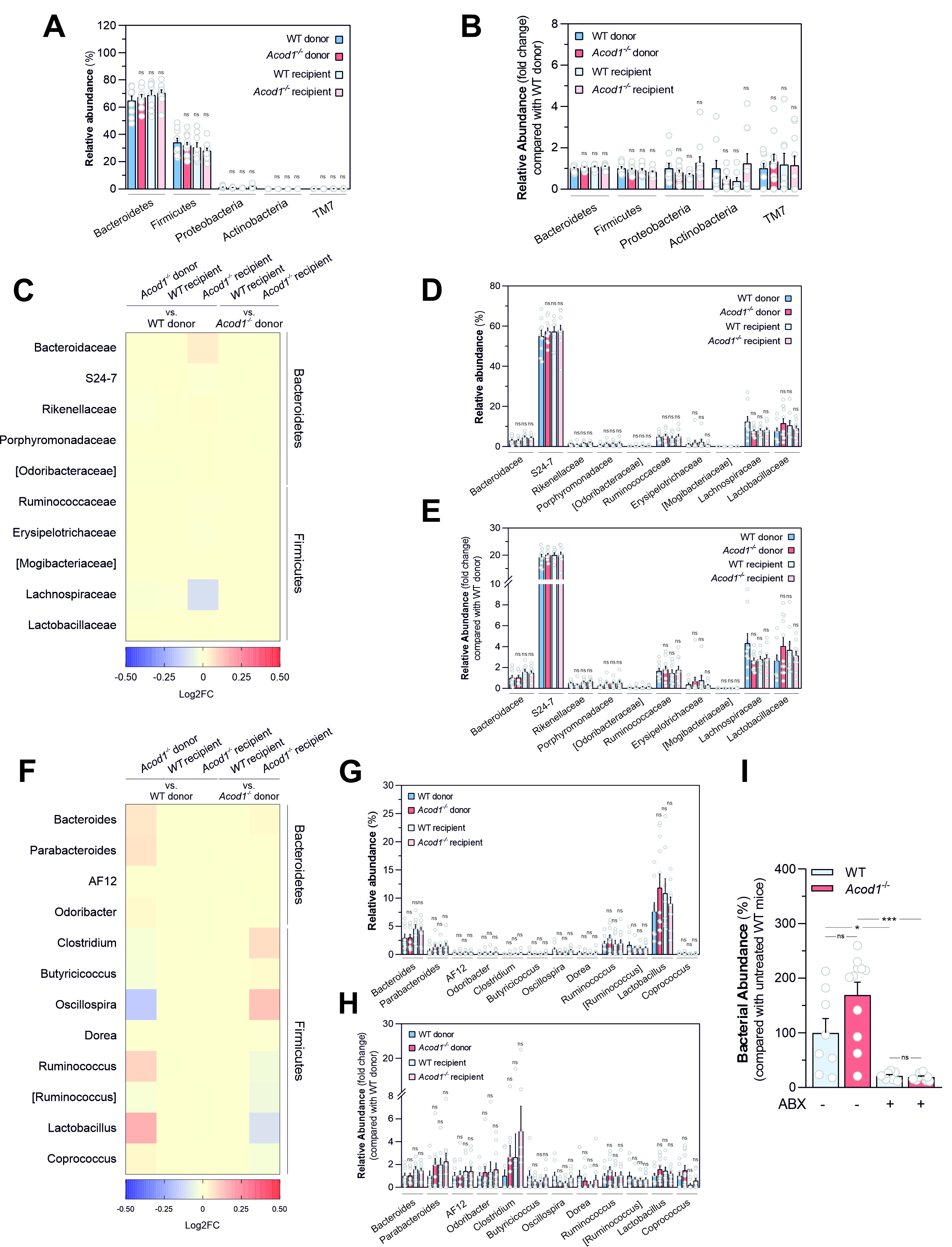

Supplement: Supplementary file 10 — Supplementary Figure 9 [file 41419_2024_6483_MOESM10_ESM.jpg]

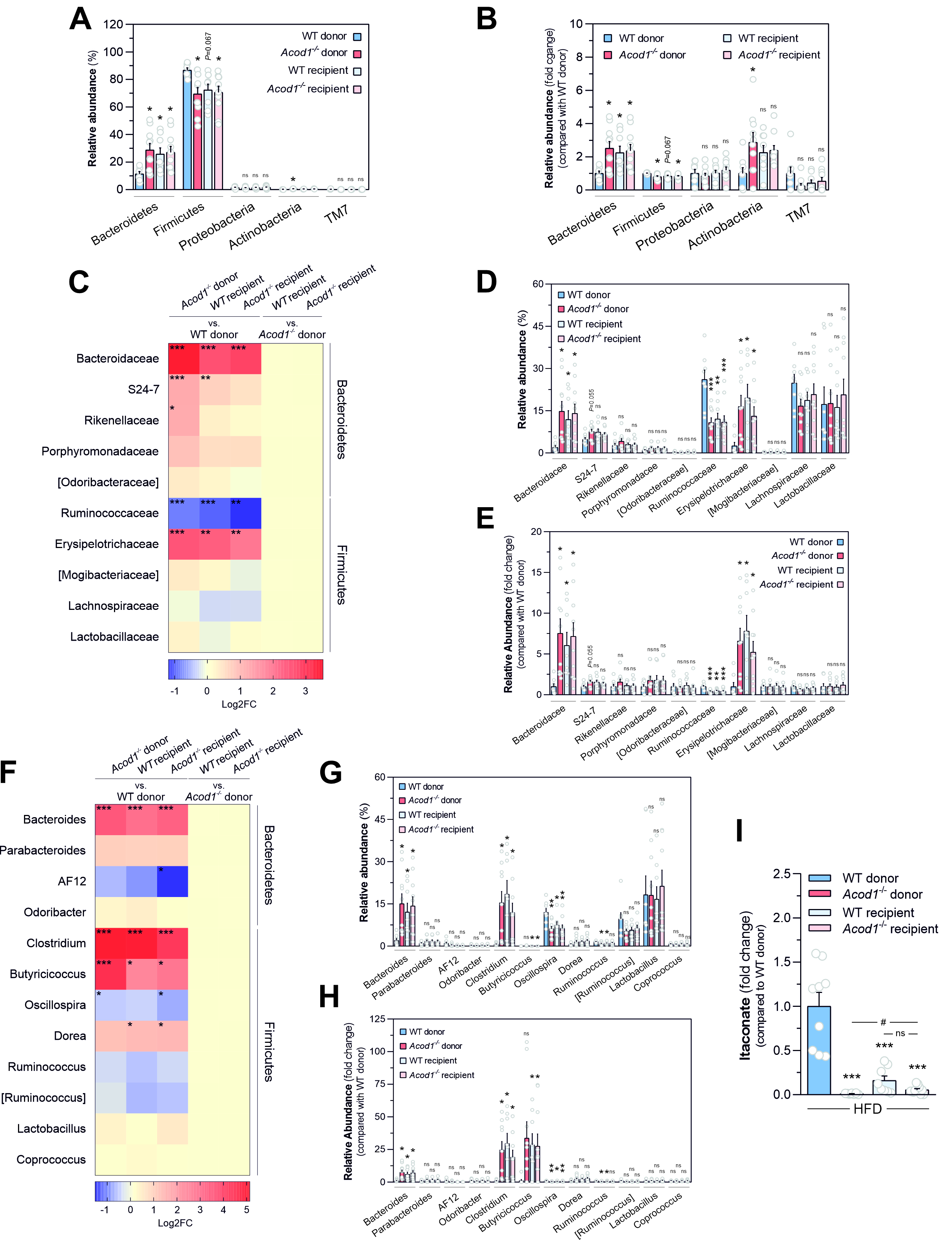

Supplement: Supplementary file 11 — Supplementary Figure 10 [file 41419_2024_6483_MOESM11_ESM.jpg]

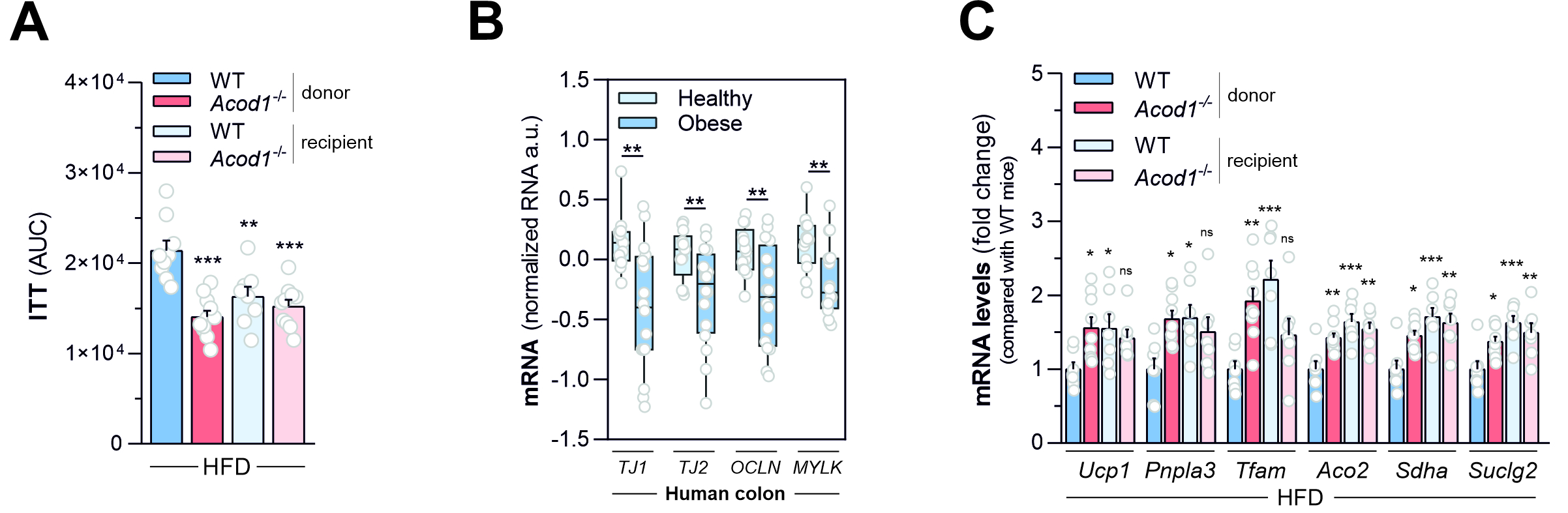

Supplement: Supplementary file 12 — Supplementary Figure 11 [file 41419_2024_6483_MOESM12_ESM.jpg]
